# A pump-free and high-throughput microfluidic chip for highly sensitive SERS assay of gastric cancer-related circulating tumor DNA via a cascade signal amplification strategy

**DOI:** 10.1186/s12951-022-01481-y

**Published:** 2022-06-11

**Authors:** Xiaowei Cao, Shengjie Ge, Weiwei Hua, Xinyu Zhou, Wenbo Lu, Yingyan Gu, Zhiyue Li, Yayun Qian

**Affiliations:** 1 Institute of Translational Medicine, Medical College, Yangzhou University, Yangzhou, 225001 People’s Republic of China; 2 College of Chemistry and Material Science, Shanxi Normal University, Linfen, 041004 People’s Republic of China; 3 Jiangsu Key Laboratory of Integrated Traditional Chinese and Western Medicine for Prevention and Treatment of Senile Diseases, Yangzhou University, Yangzhou, 225001 People’s Republic of China; 4 The First Clinical College, Dalian Medical University, Dalian, 116027 People’s Republic of China; 5 Jiangsu Key Laboratory of Experimental & Translational Noncoding RNA Research, Medical College, Yangzhou University, Yangzhou, 225001 People’s Republic of China; 6 Department of Pathology, Affiliated Hospital of Yangzhou University, Yangzhou, 225001 People’s Republic of China

**Keywords:** Catalytic hairpin assembly, Hybridization chain reaction, Surface-enhanced Raman scattering, Microfluidic chip, Circulating tumour DNA

## Abstract

Circulating tumour DNA (ctDNA) has emerged as an ideal biomarker for the early diagnosis and prognosis of gastric cancer (GC). In this work, a pump-free, high-throughput microfluidic chip coupled with catalytic hairpin assembly (CHA) and hybridization chain reaction (HCR) as the signal cascade amplification strategy (CHA–HCR) was developed for surface-enhanced Raman scattering (SERS) assays of PIK3CA E542K and TP53 (two GC-related ctDNAs). The chip consisted of six parallel functional units, enabling the simultaneous analysis of multiple samples. The pump-free design and hydrophilic treatment with polyethylene glycol (PEG) realized the automatic flow of reaction solutions in microchannels, eliminating the dependence on external heavy-duty pumps and significantly improving portability. In the reaction region of the chip, products generated by target-triggered CHA initiated the HCR, forming long nicked double-stranded DNA (dsDNA) on the Au nanobowl (AuNB) array surface, to which numerous SERS probes (Raman reporters and hairpin DNA-modified Cu_2_O octahedra) were attached. This CHA–HCR strategy generated numerous active “hot spots” around the Cu_2_O octahedra and AuNB surface, significantly enhancing the SERS signal intensity. Using this chip, an ultralow limit of detection (LOD) for PIK3CA E542K (1.26 aM) and TP53 (2.04 aM) was achieved, and the whole process was completed within 13 min. Finally, a tumour-bearing mouse model was established, and ctDNA levels in mouse serum at different stages were determined. To verify the experimental accuracy, the gold-standard qRT–PCR assay was utilized, and the results showed a high degree of consistency. Thus, this rapid, sensitive and cost-effective SERS microfluidic chip has potential as an ideal detection platform for ctDNA monitoring.

**Supplementary Information:**

The online version contains supplementary material available at 10.1186/s12951-022-01481-y.

## Introduction

Gastric cancer (GC), a heterogeneous tumour with phenotypic diversity, has become the fifth most common malignant tumour and the second leading cause of cancer-related death worldwide [1, 2]. Failure to diagnose patients in the early stages and the complicated tissue composition have resulted in the high death rate of patients with GC, and the global 5-year overall survival rate is less than 30% [3]. Therefore, there is an urgent need to develop biomarkers to assist early-stage detection and prognosis. Circulating tumour DNA (ctDNA), which can be assayed after liquid biopsy, is increasingly recognized as a noninvasive biomarker for effectively assessing the occurrence and progression of GC since it can reflect tumour burden [4–8]. TP53, which is mutated in approximately 50% of GC cases, plays important roles in regulating cell proliferation and maintaining genomic integrity and stability [9]. Moreover, the PIK3CA E542K mutation (G70271A in exon 9) is also common in GC [10]. Therefore, the sensitive detection of PIK3CA E542K and TP53 could be applied for the early diagnosis of GC. Current techniques for detecting ctDNA mainly include digital polymerase chain reaction (dPCR) and next-generation sequencing (NGS) [11, 12]. Although there have been substantial advances in detecting ctDNA using these methods, their future use is hampered by the complicated sample preparation and interference by biological environmental factors. Thus, there is great demand for the development of an accurate, sensitive and easy method for ctDNA monitoring in clinical practice.

Surface-enhanced Raman scattering (SERS) has emerged as a viable investigative tool for chemicals, biomarkers, and microorganisms due to its excellent characteristics of a narrow bandwidth and distinctive molecular fingerprint spectra, which enable the strong amplification of the Raman scattering effect [13–15]. The amplification is mainly attributed to the areas of strong electromagnetic field enhancement (also called “hot spots”), which are generated at interstitial junctions or nanogaps among metallic nanostructures through localized surface plasmon resonance (LSPR) [16]. Thus, a tremendous amount of effort has been devoted to fabricating different types of SERS-active substrates that can generate abundant “hot spots” for SERS enhancement [17–19]. In recent years, highly ordered Au nanobowl (AuNB) arrays have emerged as superior SERS-active substrates [20, 21]. Due to the special periodic pore structure, AuNB possess a large superficial area and outstanding spatial reproducibility and uniformity. Furthermore, AuNB offer a sharp plasma resonance peak and a strong interparticle near-field coupling effect, contributing to their excellent performance in biosensing and SERS applications. There has been interest in cuprous oxide (Cu_2_O) octahedra, as a p-type semiconductor, due to their narrow direct band gap of 2.2 eV and high-energy photoinduced electrons, which are specifically tailored for magnifying the Raman signal [22–24]. Under the near-field electromagnetic enhancement mechanism (EM), the E-field increases with decreasing interparticle distance. Furthermore, a chemical enhancement mechanism (CM) has also been reported: the coupling between the adsorbate and Cu_2_O octahedra could cause significant signal amplification. Hence, AuNB arrays and Cu_2_O octahedra are expected to be applied as excellent SERS-active substrates.

With the satisfactory signal enhancement by Cu_2_O octahedra and highly ordered AuNB arrays, SERS could be applied for the detection of ctDNA. However, this approach cannot meet the requirements for the early diagnosis of GC because the target ctDNA is at an ultralow level. To further enhance the detection sensitivity and more precisely identify low-abundance ctDNA, various signal amplification strategies have been introduced, including rolling circle amplification (RCA), duplex-specific nuclease (DSN), and strand-displacement amplification (SDA) [25–27]. However, most of these strategies require enzymes as catalysts and are susceptible to the sophisticated biological environment. Strand displacement-mediated DNA circuits, including catalytic hairpin assembly (CHA) and hybridization chain reaction (HCR), are typical isothermal enzyme-free signal amplification strategies. CHA is based on target recycling-oriented amplification via the displacement and hybridization of two nucleic acid hairpins and can produce numerous short double-stranded DNA (dsDNA) molecules, while HCR is achieved by cross-opening hairpins into dsDNA copolymers [28, 29]. Both these amplification strategies have been widely applied for sensing purposes; however, a single amplification strategy may still suffer from limited signal gain and insufficient sensitivity. Therefore, integrating CHA and HCR as the cascade signal amplification strategy (CHA–HCR) is expected to offer improved analytical performance [29].

Although ctDNA can be quantified sensitively by SERS through the CHA–HCR strategy, SERS-based detection still suffers from interminable manual operation in benchtop experiments, which is tedious, time-consuming and not suitable for clinical examination and diagnosis, for which portability is required [30]. Microfluidic chips, also called “lab-on-a-chip”, are promising analytical platforms with the integrated functions of sampling, reaction, separation, and detection [31, 32]. Currently, more attention has been given to integrating the SERS analytical module with microfluidic technology due to its advantages of rapid analysis, high throughput and low sample consumption [33–35]. On the one hand, SERS measurements are considerably improved because the microfluidic droplet-based method can both offer a stable and controllable reaction environment and avoid local concentration gradients and local heating phenomena. On the other hand, microfluidic platforms require detection methods appropriate for small volumes; thus, SERS microfluidics is ideal for the sensing task. Due to the low concentration of ctDNA and the complex composition of body fluids, the detection technology must have high sensitivity. In addition to the preparation of metal nanoparticles with a strong SERS effect, a simple, stable and efficient new signal amplification strategy was developed, representing a breakthrough in the development of a highly sensitive and specific detection method. Therefore, the combination of the CHA–HCR strategy with multifunctional nanomaterials and advanced detection tools provides a broader development space for the construction of new sensing platforms.

In this work, a tumour-bearing mouse model was established, and we developed a pump-free, high-throughput SERS microfluidic chip. Herein, Cu_2_O octahedra modified with two different Raman reporters, hairpin DNA 3 (hp3) and hairpin DNA 4 (hp4), were employed as the SERS probes, with the highly ordered AuNB array modified with hairpin DNA 3 (hp3) applied as the capture substrate. PIK3CA E542K and TP53, as targets, could open their corresponding hairpin DNA 1 (hp1) via complementary pairing. Then, corresponding hairpin DNA 2 (hp2) could displace the targets, forming numerous hp1–hp2 compounds. With the capillary pump, the hp1–hp2 compounds and SERS probes flowed through the microchannel and were captured by the capture substrate in sequence. One end of the hp1–hp2 compound could hybridize with hp3 on the capture substrate, and the HCR event between hp3 and hp4 on the SERS probes could be initiated, forming long nicked double-stranded DNA (dsDNA). As the reaction proceeded, an increasing number of SERS probes accumulated on the surface of the AuNB array, and numerous “hot spots” were generated, significantly enhancing the signal intensity. By analysing the linear relation between the characteristic peak intensities and target concentrations, the sensitivity of the microfluidic chip could be determined. Finally, the potential application of the proposed microfluidic chip was demonstrated by measuring the levels of PIK3CA E542K and TP53 in mouse serum at different stages. Compared with traditional detection methods, this integrated platform can process multiple samples in a single chip in parallel with high accuracy, providing a powerful tool for the early diagnosis of GC.

## Results and discussion

### Pump-free, high-throughput SERS microfluidic chip coupled with CHA–HCR strategy for ctDNA detection

**Scheme 1:**
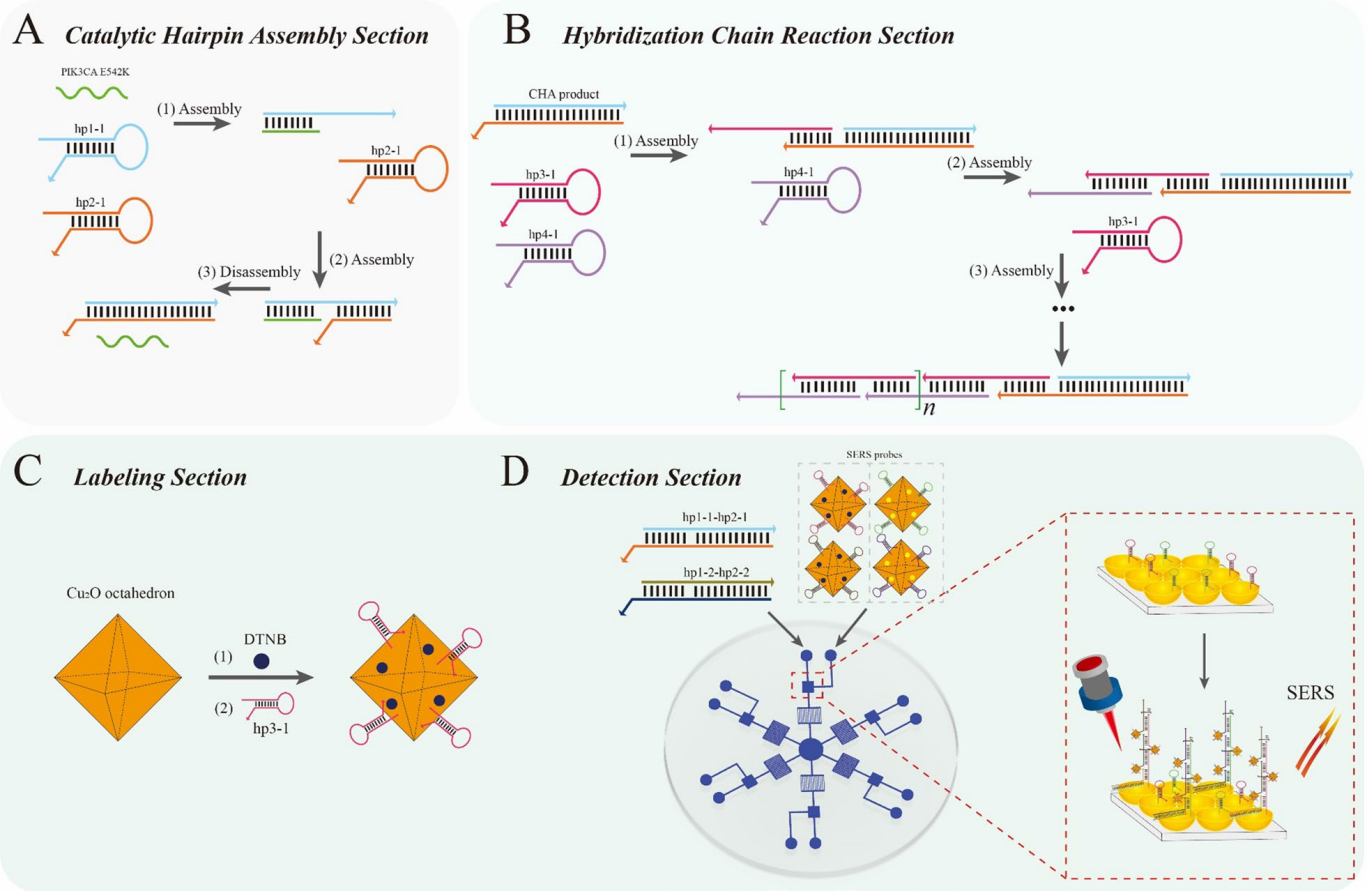
Schematic diagram of the pump-free, high-throughput microfluidic chip-based SERS assay for PIK3CA E542K and TP53. Structural mechanism of **A** CHA and **B** HCR. **C** Preparation of SERS probes and **D** detection of PIK3CA and TP53

Scheme 1 showed the working principle of the ctDNA-triggered SERS microfluidic chip. In the presence of PIK3CA E542K, hp1-1 could be opened (Scheme 1A) and then partly hybridized with PIK3CA E542K, forming an unstable dsDNA intermediate (hp1-1–PIK3CA E542K). Then, the exposed sequence of hp1-1 could assemble with hp2-1 to displace PIK3CA E542K, producing hp1-1–hp2-1 duplexes. The released PIK3CA E542K could trigger the next cycle of CHA, resulting in the generation of numerous hp1-1–hp2-1 duplexes. As shown in Scheme 1B, hp1-1–hp2-1 duplexes hybridized with hp3-1, and the exposed end of hp3-1 then hybridized with hp4-1, triggering the HCR. As a trigger, the newly exposed cohesive end of hp4-1 could open hp3-1, and the opened hp3-1 could subsequently open hp4-1. Specifically, once hp3-1 was opened, HCR occurred between hp3-1 and hp4-1, leading to the formation of long nicked dsDNA. The reaction principle for TP53 was similar. DTNB and hp3-1 (or hp4-1) could be modified onto the Cu_2_O octahedra surface via Cu–S bonds (Scheme 1C).

Here, a high-throughput SERS microfluidic chip was developed with Cu_2_O octahedra and an AuNB array as the SERS-active substrate and CHA–HCR as the dual-amplification strategy (Scheme 1D). Initially, hp1-1–hp2-1 (or hp1-2–hp2-2) duplexes and SERS probes (Cu_2_O@DTNB@hp3-1, Cu_2_O@DTNB@ hp4-1, Cu_2_O@4-ATP@ hp3-2 and Cu_2_O@4-ATP@hp4-2) were successively added to the liquid inlet. When the channel was micron- or submicron-sized, the fluid can flow at a low Reynolds number, and the viscous force is greater than the inertia force, thereby eliminating turbulence. Thus, the surface tension can become the main driving force of the fluid flow, realizing automatic flow of the reaction fluid. With a capillary pump, the sample flowed automatically to the reaction region, and the HCR was triggered, forming long nicked dsDNA with attached Cu_2_O octahedra. Due to the aggregation of Cu_2_O octahedra caused by HCR events on the surface of the AuNB array, abundant “hot spots” were generated, significantly enhancing the SERS signal intensity. Then, PIK3CA E542K and TP53 could be detected by monitoring the SERS signal in the reaction region. The SERS signal was positively correlated with the target concentration; therefore, this proposed strategy could be applied to determine the levels of PIK3CA E542K and TP53.

### Characterization of Cu_2_O octahedra

**Fig. 1:**
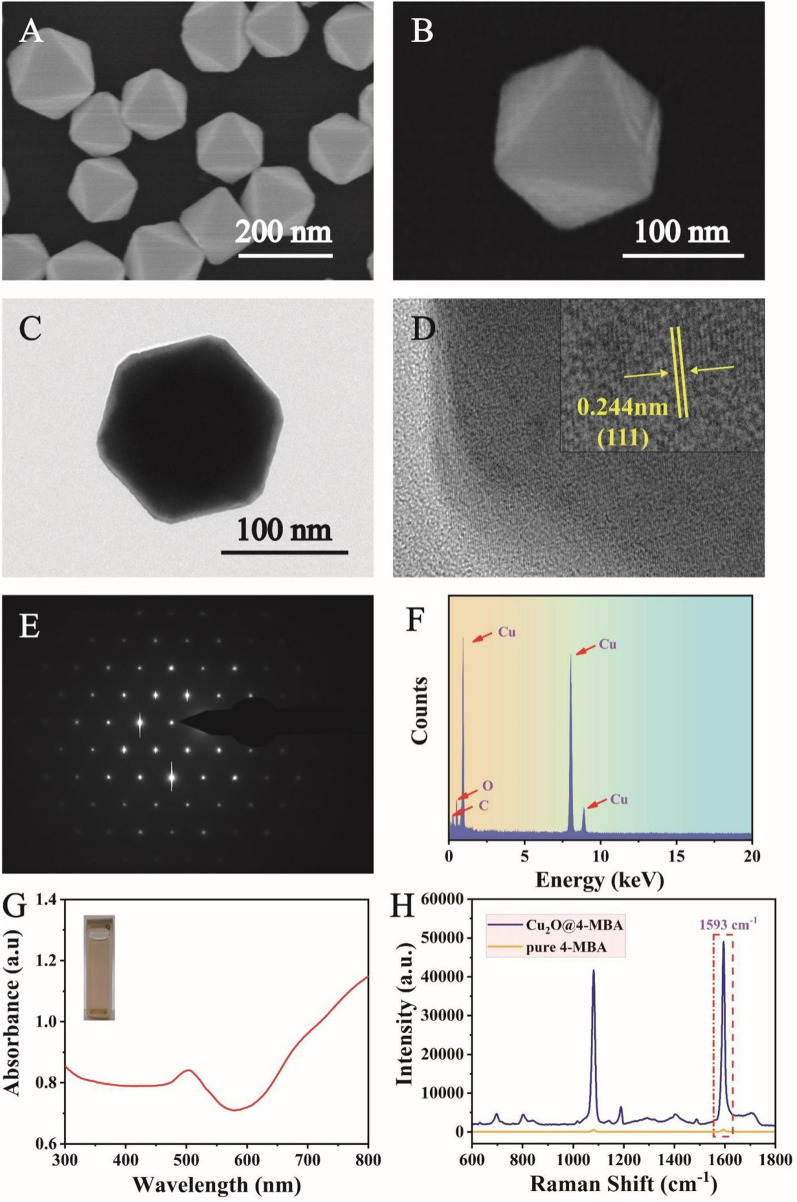
SEM images of Cu_2_O octahedra at different magnifications: **A** low magnification and **B** high magnification. Representative **C** TEM image and **D** HRTEM images of Cu_2_O octahedra. **E** SAED images, **F** EDS spectrum and **G** UV–Vis–NIR absorption spectrum of Cu_2_O octahedra. **H** SERS spectra of 4-MBA-labelled Cu_2_O octahedra and pure 4-MBA

The size and morphology of the prepared Cu_2_O octahedra were characterized by SEM and TEM. As shown in Fig. 1A and B, SEM images clearly illustrated that the morphology of Cu_2_O octahedra was highly uniform, and the average size was 130 nm. Moreover, Cu_2_O octahedra presented excellent dispersion. Figure 1C showed the TEM images of Cu_2_O octahedra, and the size corresponded to that in Fig. 1A and B. To further study the structure of Cu_2_O octahedra, HRTEM and SAED patterns were obtained (Fig. 1D and E). The HRTEM images revealed an interplanar spacing of 0.244 nm, which corresponds to the (111) facet, and the SAED pattern implied single crystalline Cu_2_O octahedra. Furthermore, the EDS analysis in Fig. 1F clearly revealed that Cu_2_O octahedra were made of Cu and O. The UV–Vis–NIR absorption spectra (Fig. 1G) showed that Cu_2_O octahedra have an obvious peak at ~ 507 nm. The plasma state of individual particle was similar to the electron state of atoms [36]. When particles aggregate, these strong interactions of electron states could form cluster states, similar to molecular orbitals formed by linear combinations of atomic orbitals. In other words, the plasma coupling model was based on the strong interactions between surface charges of particles in the aggregate, resulting in the enhancement of LSPR. To understand the SERS activity of Cu_2_O octahedra, the same experimental conditions were applied to measure the SERS spectra of 4-MBA-labelled Cu_2_O octahedra (1 × 10^− 6^ M) and pure 4-MBA (1 × 10^− 2^ M). The characteristic peak of 4-MBA at 1593 cm^− 1^ was attributed to axial deformation [37]. As shown in Fig. 1H, the intensity of 4-MBA-labelled Cu_2_O octahedra was much higher than that of pure 4-MBA. Then, the enhancement factor (EF) was calculated to determine the SERS enhancement capacity of Cu_2_O octahedra according to the equation EF = (I_SERS_/C_SERS_)/(I_RS_/C_RS_), where I represents the peak intensity at 1593 cm^− 1^, and C represents the concentration of 4-MBA. When C_SERS_ and C_RS_ were set to 1 × 10^− 6^ M and 1 × 10^− 2^ M, respectively, the EF was calculated to be 5.7 × 10^6^, suggesting that Cu_2_O octahedra could achieve satisfactory SERS enhancement. These results indicated that the prepared Cu_2_O octahedra have potential as excellent candidates for SERS sensing due to their outstanding characteristics.

### Characterization of highly ordered AuNB array

**Fig. 2:**
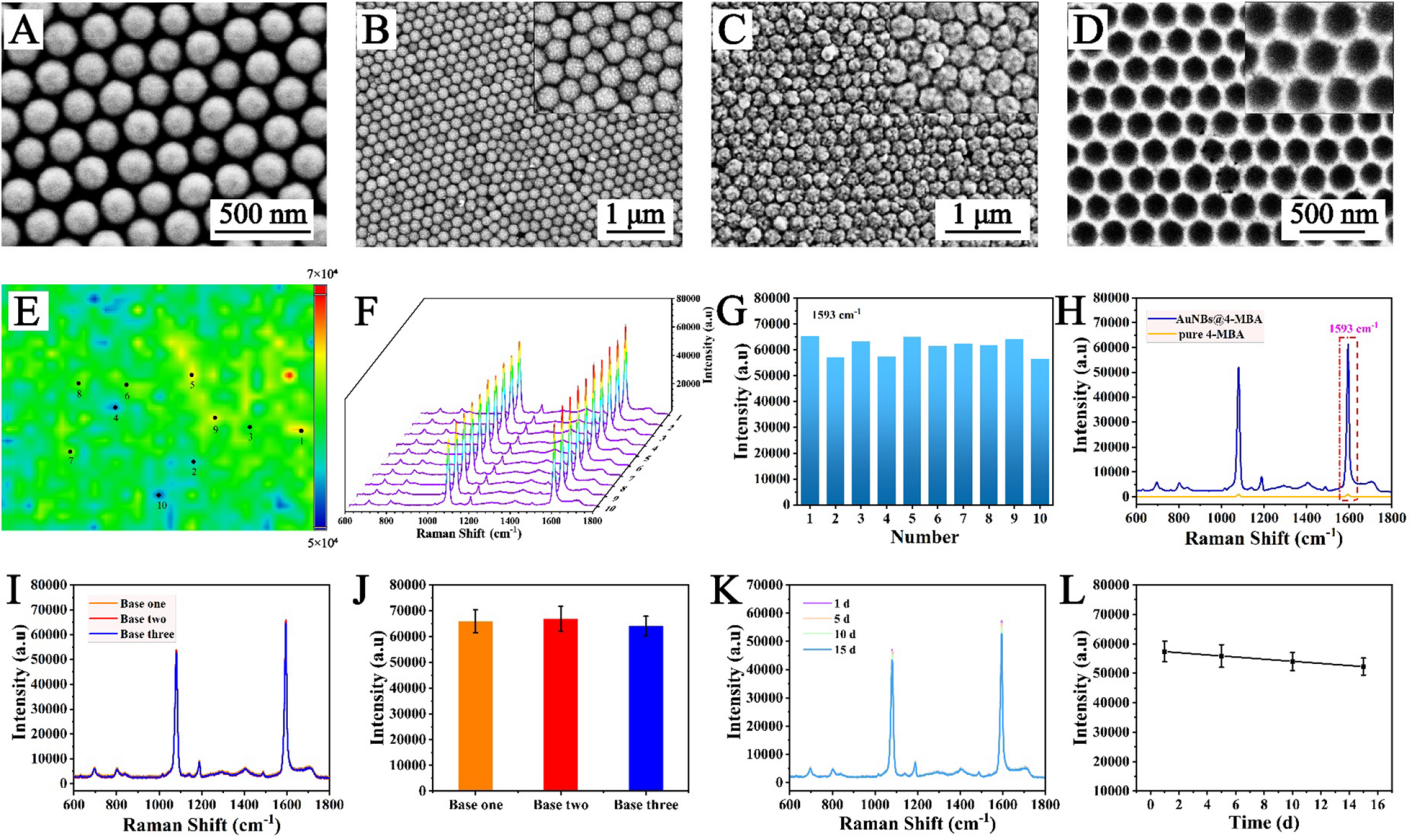
SEM images of **A** SiO_2_ colloidal crystal film, **B** SiO_2_/GNP array, **C** GNS array and **D** highly ordered AuNB array. **E** SERS mapping of the AuNB array modified with 4-MBA (1 × 10^− 8^ M). **F** SERS spectra of 10 randomly selected points on the surface of the 4-MBA-labelled AuNB array and **G** the corresponding scatter diagram of the intensity at 1593 cm^− 1^. **H** SERS spectra of AuNB arrays modified with 4-MBA (1 × 10^− 8^ M) and of pure 4-MBA (1 × 10^− 2^ M). **I** SERS spectra of 4-MBA-labelled AuNB arrays prepared in different batches and **J** the corresponding histogram. **K** SERS spectra of 4-MBA-labelled AuNB arrays prepared in the same batch and stored for different time and **L** the corresponding line chart

The AuNB array was prepared according to Additional file 1: Scheme S1. Figure 2A clearly shows large, uniform areas of SiO_2_ (~ 200 nm) that were closely packed on the slide. The SiO_2_/GNP array in Fig. 2B demonstrated that GNPs were uniformly adsorbed onto the SiO_2_ surface. As the GNPs produced by the reduction reaction between H_2_O_2_ and the growth solution were continuously deposited on the SiO_2_ surface, this SiO_2_ colloidal crystal film surface could be completely covered, forming a gold nanoshell (GNS) array. As presented in Fig. 2C, each gold shell was packed tightly in a hexagonal shape. Figure 2D shows SEM images of the AuNB array, which indicated that the thickness of each pore edge was approximately 25 nm and that the pore size was approximately 200 nm, which corresponded to the size of the SiO_2_ microspheres. To study the uniformity of the AuNB array, SERS mapping was performed on the surface of the 4-MBA-labelled AuNB array, and the scanning range was 50 × 50 mm (Fig. 2E). The results indicated that the prepared AuNB array possessed excellent homogeneity with relative standard deviation (RSD) calculated to 9.18%. Moreover, 10 different spots were selected randomly on the AuNB array, and SERS spectra were recorded (Fig. 2F). These findings revealed no obvious difference in shape between spectra, with only a slight difference in intensity (Fig. 2G). Thus, the AuNB array was a highly ordered SERS substrate with outstanding homogeneity.

To assess the SERS enhancement effect of the AuNB array, SERS spectra of pure 4-MBA (1 × 10^− 2^ M) and the 4-MBA-modified AuNB array (1 × 10^− 8^ M) were measured (Fig. 2H). The result clearly revealed the great SERS activity of the AuNB array. When C_SERS_ and C_RS_ were set to 1 × 10^− 8^ M and 1 × 10^− 2^ M, respectively, the EF was calculated to be 7.2 × 10^8^, suggesting that the AuNB array is an excellent SERS-active substrate. To verify the electromagnetic (EM) contribution, a known major mechanism of the SERS effect, of the AuNB array, a finite difference time domain (FDTD) simulation was employed (Additional file 1: Fig. S1), and the nanostructure was modelled corresponding to the architectural morphology provided by the SEM images in Fig. 2D. The results revealed that numerous “hot spots” distributed around the edges of each pore played a decisive role in SERS enhancement. The reproducibility was also studied: SERS measurements were obtained for three AuNB array substrates prepared in different batches (Fig. 2I, J), and the RSD of the peak intensities was calculated to be 7.8%. Subsequently, four AuNB arrays prepared in the same batch were stored in air for different times (1 d, 5 d, 10 d and 15 d), and the results (Fig. 2K and L) indicated robust stability of the AuNB arrays, with an RSD of 6.4%. All the outstanding characteristics of the AuNB array support its potential as a SERS substrate for the ultrasensitive detection of ctDNA.

### Feasibility evaluation and parameters optimization

**Fig. 3:**
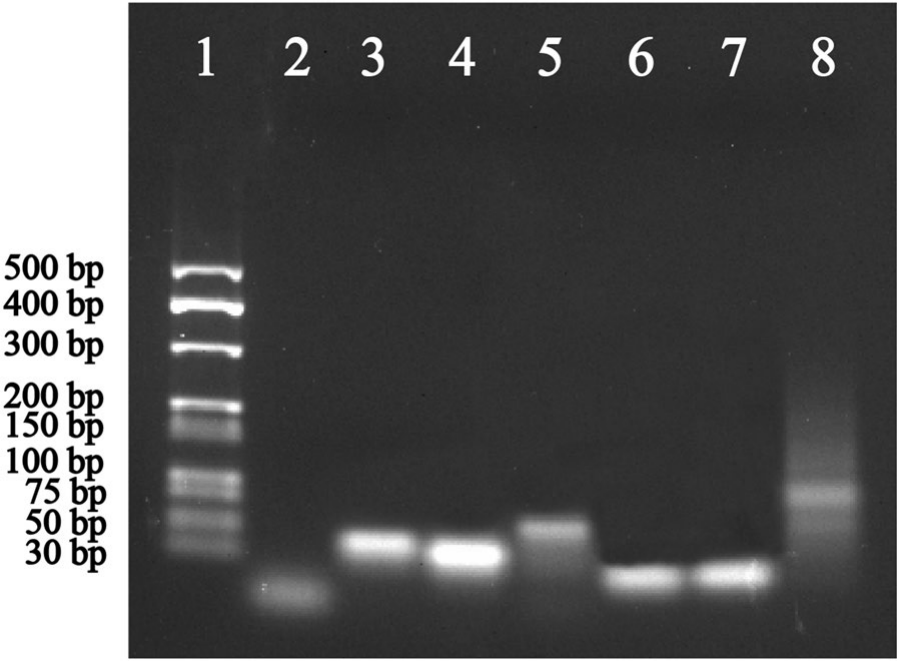
Agarose gel electrophoresis of CHA–HCR amplification products. Lane 1: marker; Lane 2: target (PIK3CA E542K); Lane 3: hp1-1; Lane 4: hp2-1; Lane 5: CHA product; Lane 6: hp3-1; Lane 7: hp4-1; Lane 8: HCR product

Before application to quantify PIK3CA E542K and TP53, the feasibility of the CHA–HCR strategy must be evaluated. Thus, agarose gel electrophoresis (Fig. 3) was employed to analyse the amplification process. An obvious high band was observed after mixing hp1-1, hp2-1 and PIK3CA E542K, indicating the successful occurrence of CHA (see Lane 5). When hp3-1 and hp4-1 were introduced to the CHA product, numerous higher bands were detected (Lane 8). Thus, the CHA product could be applied to trigger the HCR, proving the rationality of the CHA–HCR amplification strategy.

**Fig. 4:**
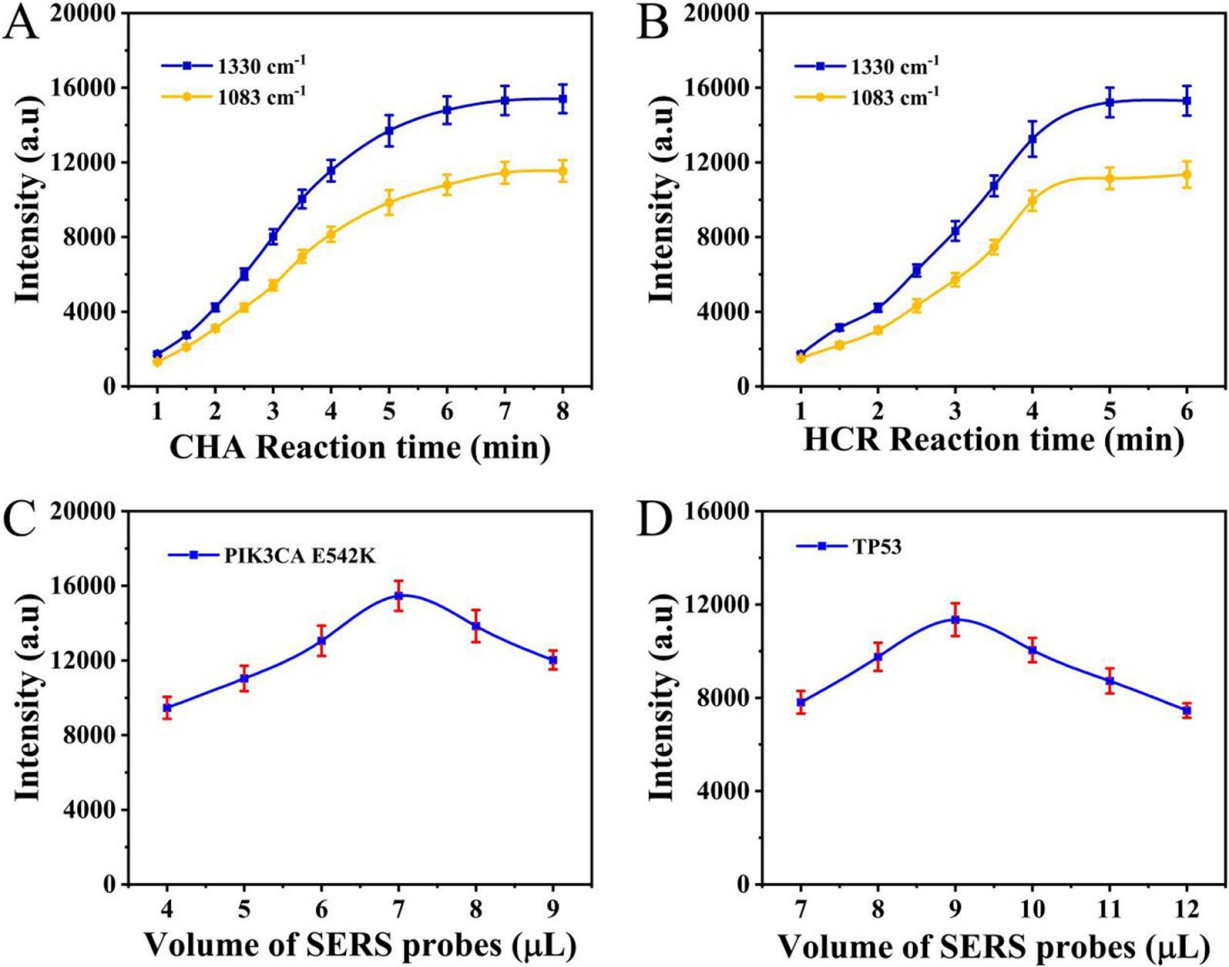
Optimization of experimental parameters: **A** CHA time; **B** HCR time; and volume of SERS probes for **C** PIK3CA E542K and **D** TP53

To develop the best target assay, several experimental parameters were investigated. The SERS intensities at 1083 cm^− 1^ and 1330 cm^− 1^ were related to the C-S stretching vibrations of 4-ATP and the C-N stretching vibrations of DTNB, respectively [38, 39]. As two important parameters for signal amplification, the incubation times for the CHA and HCR steps were optimized. Figure 4A shows that the signal gradually increased from 1 to 7 min and then remained at nearly the same level at 8 min, indicating that the optimal time for CHA was 7 min. Similarly, the time for the HCR was adjusted to 5 min (Fig. 4B). Thus, the total reaction time could be optimized to 12 min. Then, the whole detection process including the amplification reaction and the acquisition time of SERS signals could be finished within 13 min. Moreover, the volumes of the SERS probes for PIK3CA E542K and TP53, two other key parameters, were also investigated. As shown in Fig. 4C, the SERS intensity at 1330 cm^− 1^ significantly increased with increasing volume of the SERS probe for PIK3CA E542K over the range 4–7 µL and then showed a downwards trend as the volume continued to increase. This result was probably because a high volume of the SERS probe for PIK3CA E542K could result in inadequate hybridization between hp3-1 and hp4-1; this may increase background signal, leading to decreased observed signal responses. Furthermore, steric hindrance by the bulky HCR product could hinder recognition and binding, thereby reducing the signal [40, 41]. Thus, the volume of the SERS probe for PIK3CA E542K was set to 7 µL. The volume of the SERS probe for TP53 was optimized to 9 µL according to the same approach (Fig. 4D).

### Preparation of the microfluidic chip

**Fig. 5:**
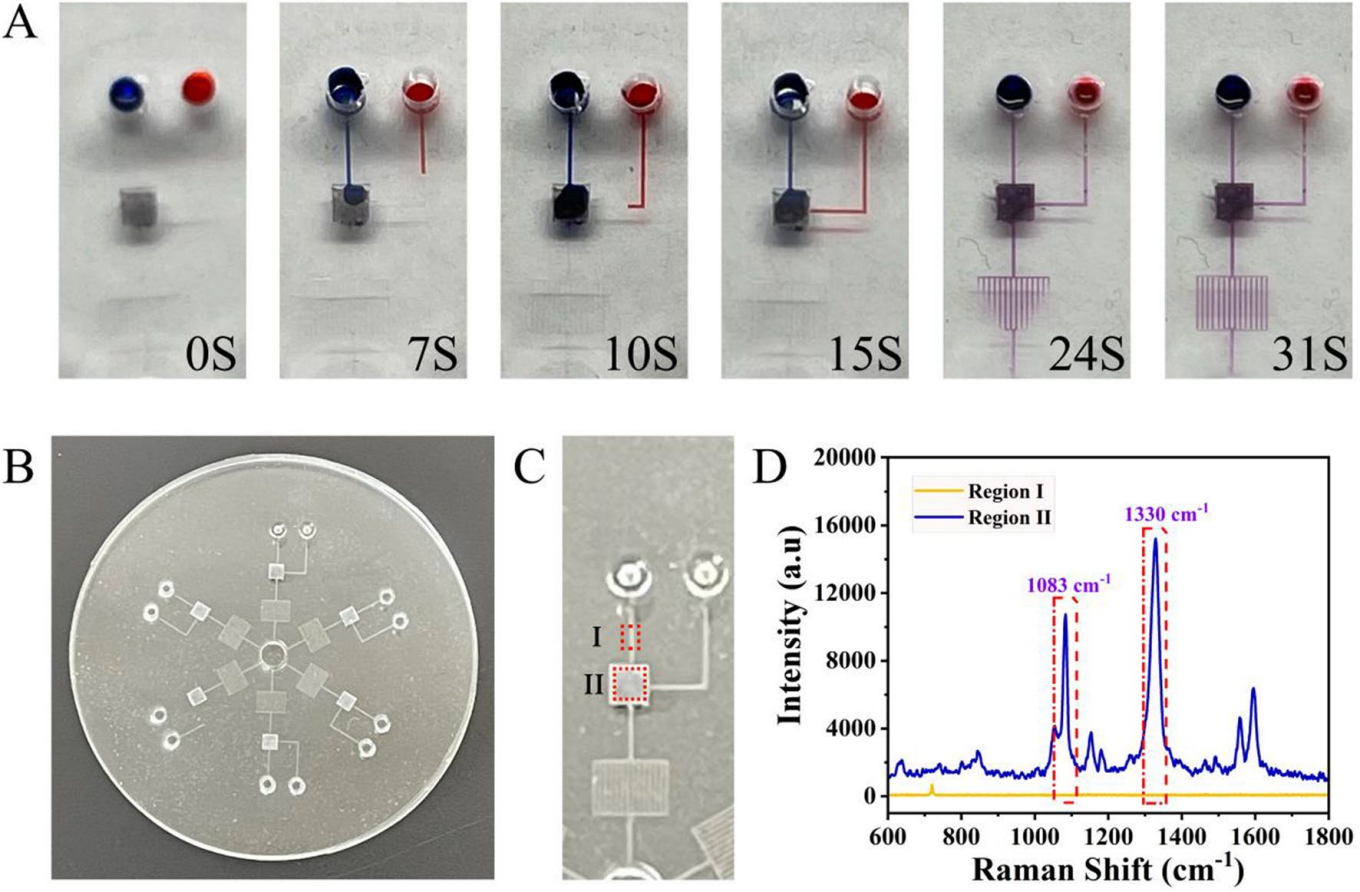
Performance evaluation of the microfluidic chip. **A** Digital images of automatic ink flow in microchannels over time. **B** Digital images of the microfluidic chip applied for target detection. **C** Two selected regions on the chip and **D** the corresponding SERS spectra

After assessing experimental feasibility and optimizing parameters, the pump-free and high-throughput microfluidic chip was fabricated, and the detailed geometry of the microfluidic device is shown in Additional file 1: Fig. S2. To ensure that the proposed microfluidic chip could be successfully applied for the analysis of targets, several performance parameters were assessed. PEG coating was employed to create a hydrophilic microchannel (experimental section, supporting information). Then, blue and red inks were used as the reaction solution flowing in the microchannel, and the flow time was measured to evaluate the effect of the hydrophilic coating. As shown in Fig. 5A, driven by the capillary pump, the inks could flow automatically in the microchannel and could outflow from the microchannel within 31 s, indicating excellent hydrophilicity. Moreover, no leakage was observed during the experiment; thus, the whole treatment process was satisfactory, proving that the proposed microfluidic chip could realize automatic liquid delivery without reliance on external pumps. Furthermore, the stability of the hydrophilic coating was excellent (Additional file 1: Fig. S3) and met the requirements for practical application.

Since the proposed microfluidic chip was made of PDMS, which is known to have its own Raman signal, it was necessary to verify whether PDMS will affect the detection results. As shown in Fig. 5C, two different regions of the microfluidic chip were selected (Fig. 5B): (I) the microchannel for the CHA product and (II) the reaction region. Since only the CHA product and PDMS were present in region I, the Raman signal recorded in this region was produced by PDMS. In addition, the Raman signal recorded in region II was produced by PDMS and the Raman reporters (DTNB and 4-ATP) modified on the surface of Cu_2_O octahedra on the HCR product. Figure 5D clearly shows that the PDMS signal was nearly null compared to that of the Raman reporters; thus, PDMS did not affect target detection. As shown in Additional file 1: Fig. S4, the microfluidic chip was used for the qualitative analysis of PIK3CA E542K and TP53, and the results were satisfactory. Therefore, the proposed pump-free, high-throughput microfluidic chip was fabricated successfully.

### Specificity and sensitivity of the SERS microfluidic chip

**Fig. 6:**
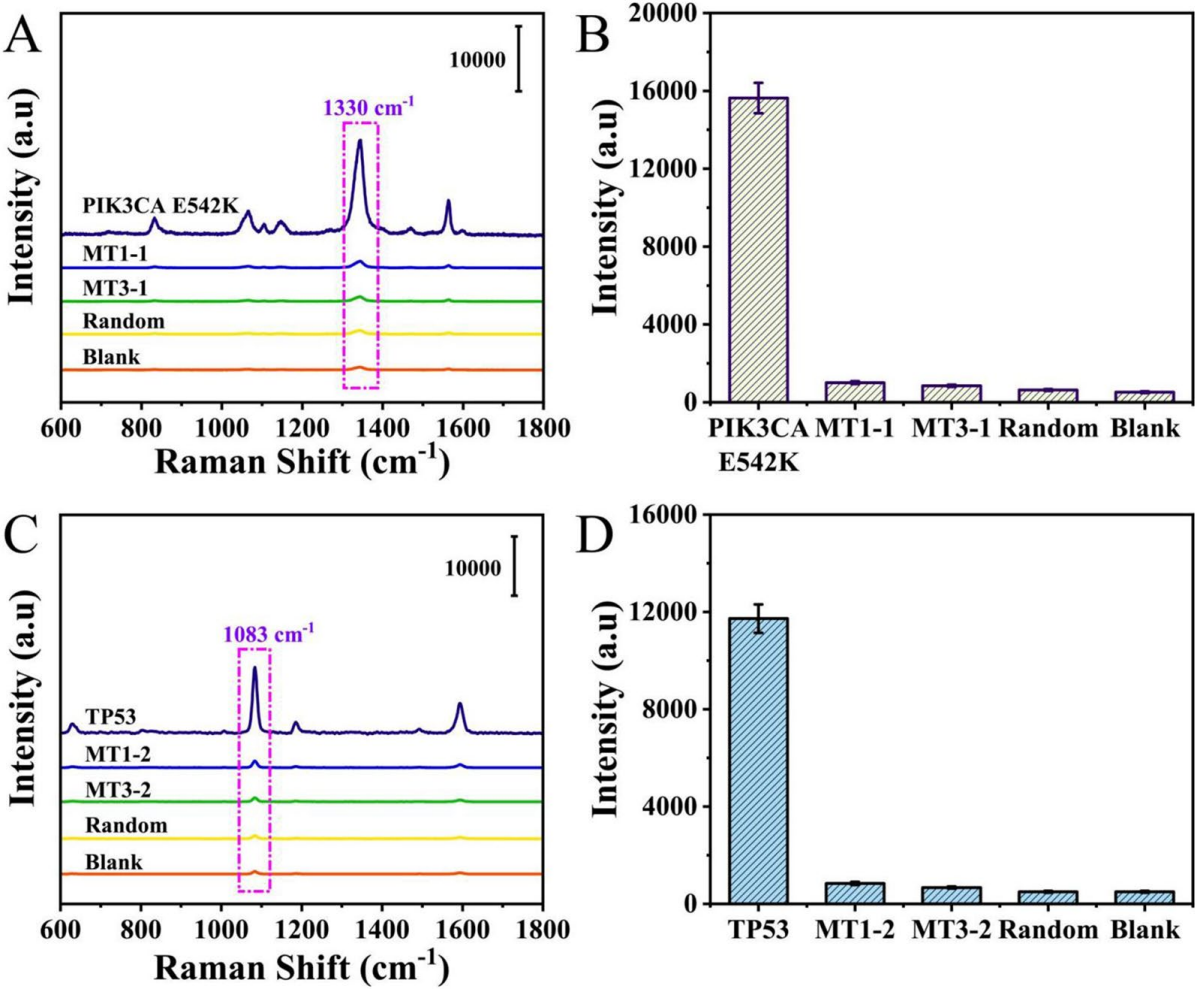
Specificity assessment of the SERS microfluidic chip. **A** SERS spectra of PIK3CA E542K, MT1-1, MT3-1, the random sequence and the blank control. **B** Corresponding histogram of peak intensities at 1330 cm^− 1^. **C** SERS spectra of TP53, MT1-2, MT3-2, the random sequence and the blank control. **D** Corresponding histogram of peak intensities at 1083 cm^− 1^

Specificity was considered the most crucial parameter for practical application, and it was necessary to evaluate the specificity of our proposed SERS microfluidic chip. Thus, one-base mismatch (MT1-1 and MT1-2), three-base mismatch (MT3-1 and MT3-2) and random sequences were introduced as interference to verify the specificity under the optimized experimental conditions. As shown in Fig. 6A, products after the CHA reaction and SERS probes (Cu_2_O@DTNB@hp3-1 and Cu_2_O@DTNB@ hp4-1) were added, and the SERS spectra demonstrated that PIK3CA E542K showed much higher intensity than the interference. The intensities at 1330 cm^− 1^ shown in Fig. 6B clearly demonstrated that the SERS microfluidic chip could accurately distinguish PIK3CA E542K from the interference. Similarly, products after the CHA reaction and SERS probes (Cu_2_O@4-ATP@hp3-2 and Cu_2_O@4-ATP@ hp4-2) were added, and the results proved that the specificity for TP53 was also excellent (Fig. 6C, D). Furthermore, the sensitivity of the proposed microfluidic chip was also assessed (Additional file 1: Fig. S6) and the results clearly demonstrated that the detection performance of the chip for PIK3CA E542K and TP53 was satisfactory in the range from 10 aM to 100 pM, with the LOD calculated to 1.26 aM and 2.04 aM in serum respectively which was much lower than that of other recent publications (Table 1). When compared with our previous publications, this developed microfluidic chip showed better performance in multiple aspects, such as signal uniformity and high-throughput screening although the LOD was similar and the detection time was a little bit longer. Thus, this proposed microfluidic chip could serve as an excellent platform for ctDNA monitoring.

**Table 1:** Comparison of the proposed strategy with other reported methods

Method	Analyte	Time	Detection range	LOD (M)	Ref.
Electrochemistry	miRNA-155	3 h	1 × 10^− 16^–1 × 10^− 8^ M	3.96 × 10^− 17^	[4]
Fluorescence	miRNA-21	70 min	1 × 10^− 14^–1 × 10^− 9^ M	8.1 × 10^− 15^	[5]
Fluorescence	E542K-ds-ctDNA	250 min	1 × 10^− 12^–1 × 10^− 10^ M	3.161 × 10^− 13^	[6]
SERS	miRNA-21	60 min	3.3 × 10^− 16^–3.3 × 10^− 12^ M	4.2 × 10^− 17^	[7]
SERS	BRAF V600E	5 min	1 × 10^− 17^–1 × 10^− 10^ M	3 × 10^− 17^	[8]
SERS	PIK3CA E542K	13 min	1 × 10^− 17^–1 × 10^− 10^ M	1.26 × 10^− 18^	This work
	TP53			2.04 × 10^− 18^	

### Characterization of the tumor-bearing mouse model

**Fig. 7:**
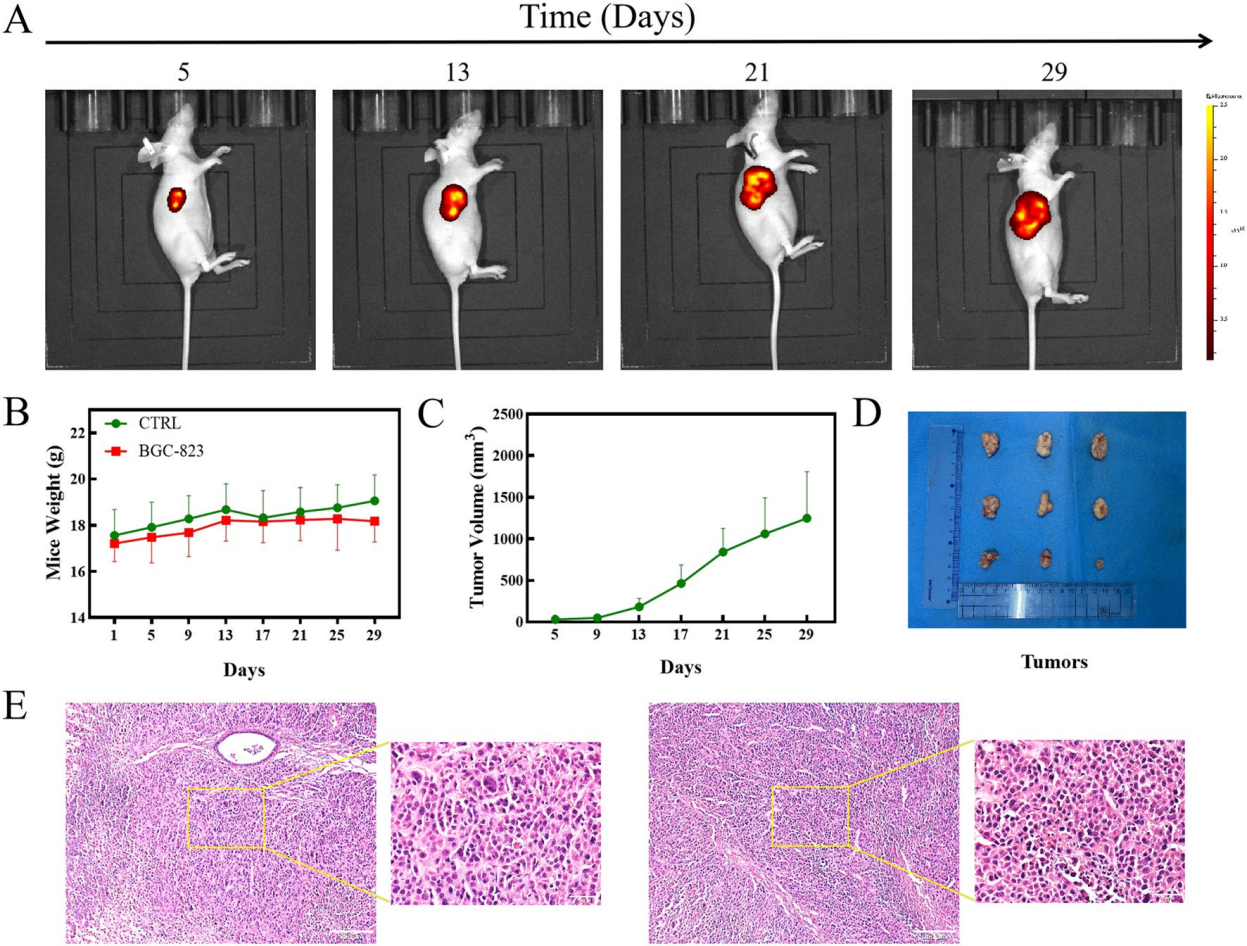
Solid gastric cancer growth in nude mice. **A** Nude mice with gastric cancer. **B** The weight of nude mice. **C** Tumour volume curves. **D** Xenograft tumour volume and HE staining

The growth of subcutaneous tumours was observed and recorded using a live animal imaging system (PerkinElmer, USA) (Fig. 7A and Additional file 1: Fig. S5). Mouse weight was monitored continuously throughout the experiment. Compared with the normal control group, the transplanted tumour group did not show a significant difference in body weight (Fig. 7B). The tumour volume curves of the nude mice are shown in Fig. 7C and D. Haematoxylin-eosin (HE) staining of tumour tissue was performed, revealing that the cells were disordered, with a nonuniform size and enlarged nucleus (Fig. 7E). Since a subcutaneous tumour appeared in one mouse (Additional file 1: Fig. S5 NO. 9) later than those in the other mice in the GC group, the data for this mouse were excluded from subsequent analyses.

### Application in real samples

**Fig. 8:**
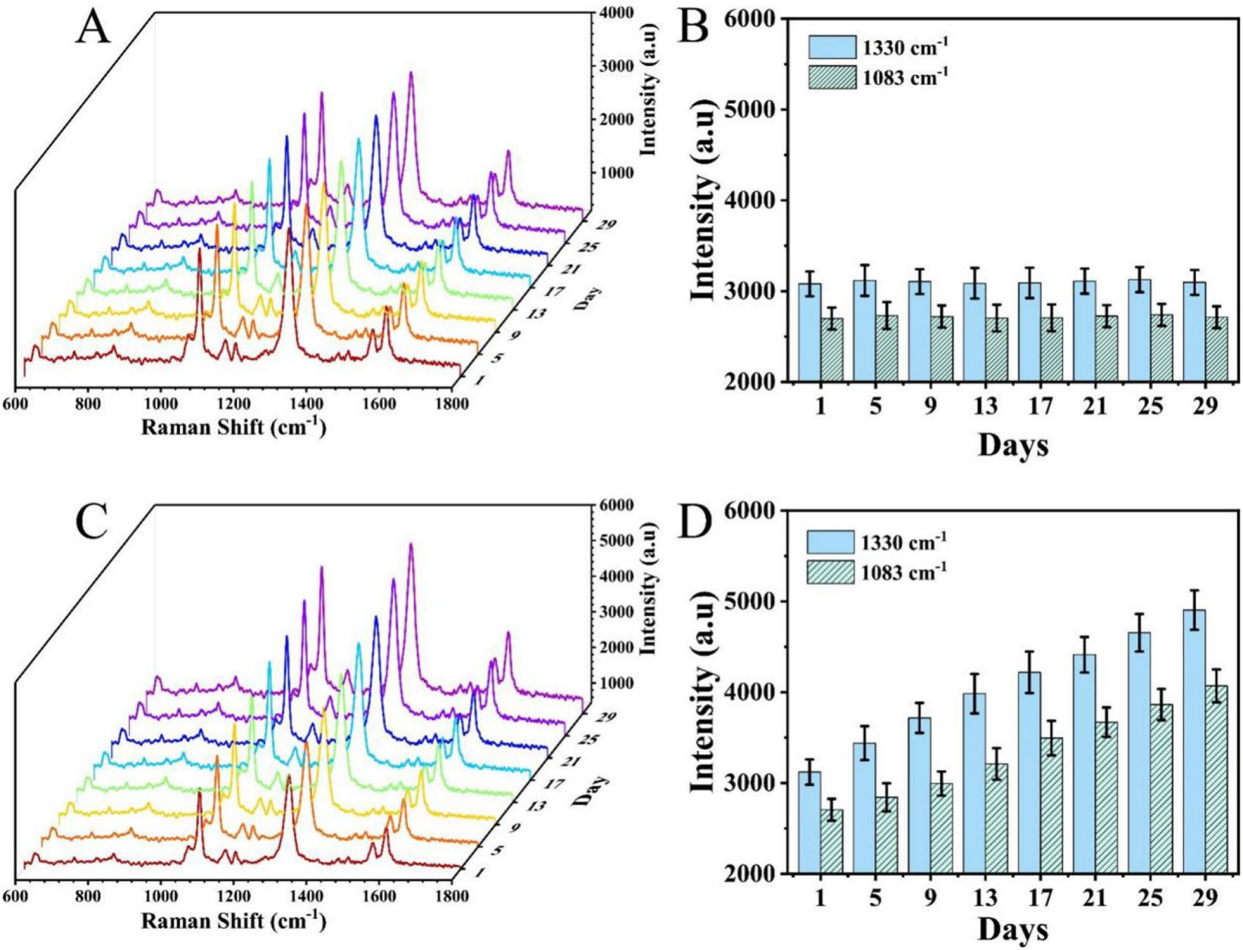
Application potential in preclinical samples. **A** SERS spectra of PIK3CA E542K and TP53 in healthy controls. **B** Corresponding histogram of intensities at 1083 cm^− 1^ and 1330 cm^− 1^. **C** SERS spectra of PIK3CA E542K and TP53 in tumour-bearing mouse serum. **D** Corresponding histogram of intensities at 1083 cm^− 1^ and 1330 cm^− 1^

After a tumour-bearing mouse model was established, the proposed SERS microfluidic chip was applied for the quantitative determination of PIK3CA E542K and TP53 in PBS and serum (Additional file 1: Fig. S6). To further investigate the clinical application of the proposed method, SERS was applied to analyse the levels of PIK3CA E542K and TP53 in mouse serum obtained at different stages of tumour development (1, 5, 9, 13, 17, 21, 25 and 29 d). Additional file 1: Fig. S7 presents the spectra of all eight tumour-bearing mice, and the peak intensities at 1330 cm^− 1^ and 1083 cm^− 1^ were substituted into the corresponding linear regression equation with the ctDNA concentrations recorded in Additional file 1: Tables S3–S10. Figure 8A and C show the average SERS spectra, which demonstrated that the level increased as the tumour progressed. The corresponding histograms in Fig. 8B and D indicated that this method could distinguish the levels in mice at different stages. To verify the accuracy of the results, qRT–PCR was employed (Table 2). The results generated by SERS were consistent with those by qRT–PCR, proving the excellent accuracy of our proposed method. These results clearly indicate that our proposed microfluidic chip is suitable for the accurate detection of ctDNA in real samples.

**Table 2:** Average results of SERS qRT–PCR results for real samples

Day	SERS (fM)		qRT–PCR (fM)		Relative error (%)	
	PIK3CA E542K	TP53	PIK3CA E542K	TP53	PIK3CA E542K	TP53
1	0.214	0.198	0.204	0.188	4.67	5.05
5	0.333	0.304	0.317	0.289	4.80	4.93
9	0.466	0.396	0.443	0.376	4.94	5.05
13	0.669	0.542	0.637	0.514	4.78	5.17
17	0.891	0.839	0.843	0.797	5.38	5.01
21	1.143	1.081	1.084	1.017	5.16	5.92
25	1.556	1.455	1.476	1.377	5.14	5.36
29	2.131	1.983	2.035	1.889	4.50	4.74

## Conclusions

In summary, we developed a pump-free, high-throughput SERS microfluidic chip for the detection of trace ctDNA assisted by a cascade signal amplification strategy (CHA–HCR). In this microfluidic chip, long nicked dsDNA could be extended after CHA products triggered HCR; thus, numerous Cu_2_O octahedra could be gathered on the surface of the highly ordered AuNB array, generating abundant “hot spots” to significantly amplify the local electromagnetic field. With the CHA–HCR strategy, markedly stronger SERS signals could be achieved, and the ultrasensitive detection of PIK3CA E542K and TP53 was realized with calculated LODs of 1.26 am and 2.04 am, respectively. Furthermore, the uniformity, stability, reproducibility and specificity were satisfactory. The entire detection process finished in 13 min without dependence on external pumps, presenting great portability. The high-throughput nature of this system would allow the analysis of multiple samples in parallel. As expected, the target levels in healthy controls and tumour-bearing mouse serum were quantitatively detected with credible results. Although the development of this SERS microfluidic chip is in the initial stage, the gratifying results obtained with this method bring great hope to extend the reach of SERS towards true application in clinical diagnostics.

## Supplementary Information

**Additional file 1. **Supporting information including additional methods, figures and tables.

## Data Availability

Not applicable.
