# Supplementary material for: A pump-free and high-throughput microfluidic chip for highly sensitive SERS assay of gastric cancer-related circulating tumor DNA via a cascade signal amplification strategy
Source: J Nanobiotechnology. 2022 Jun 11;20:271. doi: 10.1186/s12951-022-01481-y (PMC9188168; doi:10.1186/s12951-022-01481-y)
Supplement: Supplementary file 1 — Additional file 1. Supporting information including additional methods, figures and tables. [file 12951_2022_1481_MOESM1_ESM.docx]

**A pump-free and high-throughput microfluidic chip for highly sensitive SERS assay of gastric cancer-related circulating tumor DNA via a cascade signal amplification strategy**

Xiaowei Cao^1,3,5^,*, Shengjie Ge^1,3,5^, Weiwei Hua^1,3,5^, Xinyu Zhou^1,3,5^, Wenbo Lu^2^, Yingyan Gu^1,3,5^, Zhiyue Li^4^ and Yayun Qian^1,3,5^,*

^1^Institute of Translational Medicine, Medical College, Yangzhou University, Yangzhou, 225001, P. R. China.

^2^Shanxi Normal University, College of Chemistry and Material Science, Linfen, 041004, P. R. China.

^3^Jiangsu Key Laboratory of Integrated Traditional Chinese and Western Medicine for Prevention and Treatment of Senile Diseases, Yangzhou University, Yangzhou, 225001, P. R. China.

^4^The First Clinical College, Dalian Medical University, Dalian, 116027, P. R. China.

^5^Jiangsu Key Laboratory of Experimental & Translational Noncoding RNA Research, Medical College, Yangzhou University, Yangzhou, 225001, P. R. China.

*Correspondence: [cxw19861121@163.com](mailto:cxw19861121@163.com) and [yyqian@yzu.edu.cn](mailto:yyqian@yzu.edu.cn)

**1. Experimental section**

**1.1 Reagents**

Potassium carbonate (K_2_CO_3_), sodium borohydride (NaBH_4_), chloroauric acid (HAuCl_4_), hydrogen peroxide (H_2_O_2_), copric chloride dihydrate (CuCl_2_∙2H_2_O), polyvinylpyrrolidone K30 (PVP), sodium hydroxide (NaOH), hydrofluoric acid (HF), methanol and bovine albumin (BSA), ascorbic acid (AA) and phosphate-buffered saline (PBS) were purchased from Sinopharm Chemical Reagent Co. Ltd. (China). Silicon dioxide (SiO_2_, MP-2040) was provided by Nissan Chemical Co. Ltd. (Japan). 5,5'-dithiobis-(2-nitrobenzoic acid) (DTNB), 4-aminothiophenol (4-ATP), polydimethylsiloxane (PDMS) and polyethylene glycol (PEG) was obtained from Sigma-Aldrich. All reagents were used as received without any further purification. Experimental water was purified via a Milli-Q water purification system (Millipore, >18 M). The real-time polymerase chain reaction (qRT-PCR) kits were purchased from GeteinBiotech (China). All involved oligonucleotides synthesized by Sangon Biotech (Shanghai, China) were listed in Table S1.

**1.2 Xenograft experiment**

Totally 15 four-week-old nude mice were obtained from the Comparative Medicine Center of Yangzhou University and approved by the Animal Care Committee of Yangzhou University. They were randomly assigned to normal control (n=6) and gastric cancer group (n=9). The human gastric cancer BGC-823 cells labeled with a red fluorescent protein (1.5×10^7^/L) were subcutaneously injected into the right flank of nude mice. The mice were weighed every four days and the tumor volume (V) was calculated as follows: V= (length×width^2^)/2. Venous bloods were obtained through the tail vein. On the 29th day, tumor tissues were stripped after unified sacrifice.

**1.3 Synthesis of Cu_2_O octahedra**

Cu_2_O octahedra applied in the experiment was prepared via the chemical precipitation-reduction method reported by Yu’s [1]. Briefly, 0.34 g CuCl_2_∙2H_2_O was mixed with 9.0 g PVP in a beaker containing 200 mL deionized water followed by adding NaOH (10 mL, 2 M) and the mixture was kept stirring at 55 ℃ for 40 min. Then AA (10 mL, 0.6 M) was added dropwise into the solution with precipitate in brick red color generated. After three hours of stirring at 55 ℃, the precipitate cooling down was purified by water and ethanol three times respectively. Finally, Cu_2_O octahedra could be obtained after drying overnight at 60 ℃.

**1.4 Preparation of SERS probes**

4-ATP and DTNB were applied as the Raman reporters. Firstly, 150 μL 4-ATP (or DTNB) at the concentration of 1 mM was mixed thoroughly with 4 mL prepared Cu_2_O octahedra solution and the mixture was stirred at room temperature (600 rpm, 50 min). In order to be modified onto the surface of Cu_2_O octahedra, the hairpin DNAs (hp3-1, hp3-2, hp4-1, hp4-2) were activated by TECP. The hairpin DNA (20 μL, 0.1 mM) was added to the newly prepared TECP (25 μL, 1 mM). Then, the activated hairpin DNA was mixed with 2 mL 4-ATP (or DTNB)-labeled Cu_2_O octahedra. Subsequently, the prepared SERS probes (Cu_2_O@DTNB@hp3-1, Cu_2_O@DTNB@hp4-1, Cu_2_O@4-ATP@hp3-2 and Cu_2_O@4-ATP@hp4-2) were washed and incubated in BSA solution (20 μL, 1w%) for 1 h to eliminate nonspecific binding. Finally, the mixture was purified by centrifugation (12,000 rpm) for three times and suspended in the PBS buffer.

**1.5 Fabrication of highly ordered AuNBs array**

The highly ordered AuNBs array used in this experiment was prepared by depositing a layer of gold nanoparticles (GNPs) on the surface of SiO_2_ colloidal crystal film by chemical reduction method and removing the SiO_2_ colloidal crystal film by HF etching (Scheme S1). The high quality and large area SiO_2_ colloidal crystal film was prepared by mature vertical evaporation technology. Firstly, 15 mL purified SiO_2_ colloidal solution with excellent monodispersity was added into the beaker and then a glass slide was placed vertically. As illustrated in Scheme S1A, the liquid level could decrease with the evaporation of the solution, and the monodisperse SiO_2_ microspheres could self-assemble on the slide by capillary driving force, finally forming a periodically arranged SiO_2_ colloidal crystal film. When the solution was about to evaporate completely, the slide with long film was taken out and dried in the air. Thus, the colloidal crystal film of SiO_2_ could be synthesized. Then, 1 mL APTES was mixed with 50 mL ethanol and the prepared SiO_2_ crystal film was inserted into the beaker for 24 h. After drying in the air, the aminoated SiO_2_ crystal film was placed in the 100 mL constantly stirring gold nanoparticles (GNPs) solution for 6 h which was prepared according to the method reported by Liu et al. [2]. Subsequently, the unreacted GNPs were eliminated and the SiO_2_/GNPs array could be obtained by electrostatic forces (Scheme S1B). Next, the prepared SiO_2_/GNPs array was placed in 100 mL K_2_CO_3_-HAuCl_4_ growth solution which was obtained by adding HAuCl_4_ (6 mL, 1%) into K_2_CO_3_ (400 mL, 0.025%). After adding H_2_O_2_ (1 mL, 3%) solution quickly and reacted for 50 min, the GNSs array could be achieved followed by washing and drying. Finally, the scotch tape was applied to stick the prepared GNSs array followed by transferring to the HF solution overnight which was prepared by adding deionized water, ethanol and HF at a volume ratio of 8:1:1. Thus, the high-ordered AuNBs array was fabricated and then the activated hairpin DNAs (hp3-1 and hp3-2) was added forming the hairpin DNAs modified AuNBs array.

**1.6 Preparation of pump-free SERS microfluidic chip**

The proposed microfluidic chip consisted of three parts: polydimethylsiloxane (PDMS) microchannel, glass slide and highly-ordered AuNBs substrate. Firstly, the structure of microfluidic chip was designed with AutoCAD software and the corresponding silicon wafer template could be prepared. 100 g PDMS was mixed with 10 g curing agent followed by stirring for 30 min. Then, the mixture was placed in a vacuum chamber to remove the air bubbles. Subsequently, the mixed solution was poured onto the template and then placed on a hot plate at 80 ℃ for 3 h. Then, puncher was applied to processed PDMS. The obtained PDMS substrate and the glass slide were soaked in ethanol and isopropyl alcohol for 5min ultrasonic cleaning followed by drying. After placing the AuNBs array on the glass slide by etching, the glass slide was laminated to PDMS and the microfluidic chip could be obtained

**1.7 Hydrophilic treatment**

To ensure the automatic flowing of the reaction solution, hydrophilic surface treatment was performed by coating PEG polymer on the chip [3]. Briefly, PDMS layer and glass slide were cleaned with isopropanol and acetone respectively for 5 min followed by drying. Then, the chip was exposed to an oxygen plasma for 90 s and an appropriate amount of PEG was measured to coat the chip followed by placing on the hot plate at 150 ℃ for 30 min. Subsequently, the microfluidic chip was washed with isopropyl alcohol and deionized water for five times respectively, and placed at 4 ℃ for 1 h. Thus, the hydrophilic surface treatment could be finished.

**1.8 SERS measurement**

In the detection process, samples were mixed with equivalent amounts (1 μM, 10 μL) of hp1-1 and hp2-1, then hp1-2 and hp2-2 (1 μM, 10 μL) were added and the mixture was reacted for 7 min to accomplish the CHA reaction. Then the CHA product (hp1-1-hp2-1 and hp1-2-hp2-2) and the prepared SERS probes (Cu_2_O@DTNB @hp3-1 and Cu_2_O@DTNB@hp4-1 (1 μM, 7 μL), Cu_2_O@4-ATP@hp3-2 and Cu_2_O@ 4-ATP@hp4-2 (1 μM, 9 μL)) were dropped into the liquid inlet with a porous pipette successively. The reaction solution could flow in the microchannel under the action of the capillary pump and the HCR reaction could happen on the surface of highly ordered AuNBs array. SERS spectra were recorded by a Raman spectrometer (Renishaw, U.K.) in the reaction region at 785 nm (5 mW) with an exposure time of 10 s and 50-objective. In order to decline the background noises of instrument on the experimental results, the measured spectra were handled with smoothing and baseline correction. For each sample, SERS measurements were performed at more than 25 randomly selected positions in the reaction region, to realize the quantification of targets and guarantee the effectiveness of the results.

**1.9 Characterization**

The size, morphology and composition were characterized by TECNAI 12 transmission electron microscope (TEM) and Hitachi S-4800 field-emission scanning electron microscope. UV-Vis-NIR absorption spectra were measured by Agilent Cary 60 UV-Vis-NIR spectrometer. FEI field emission transmission electron microscope equipped with energy-dispersive X-ray spectroscopy (EDS) was applied for the high-resolution transmission electron microscopy (HRTEM) and selected area electron diffraction (SAED) images.

**1.10 Data analysis**

The LOD was calculated based on the characteristic peaks of the SERS spectra using the following equation: $LOD=10\frac{(C_{blank}+3SD)-a}{b}$, where a and b were the variables obtained with a linear regression of the signal-concentration curve, SD was the standard deviation and C_blank_ is the SERS intensity of the blank sample.

**2. Results and discussion**


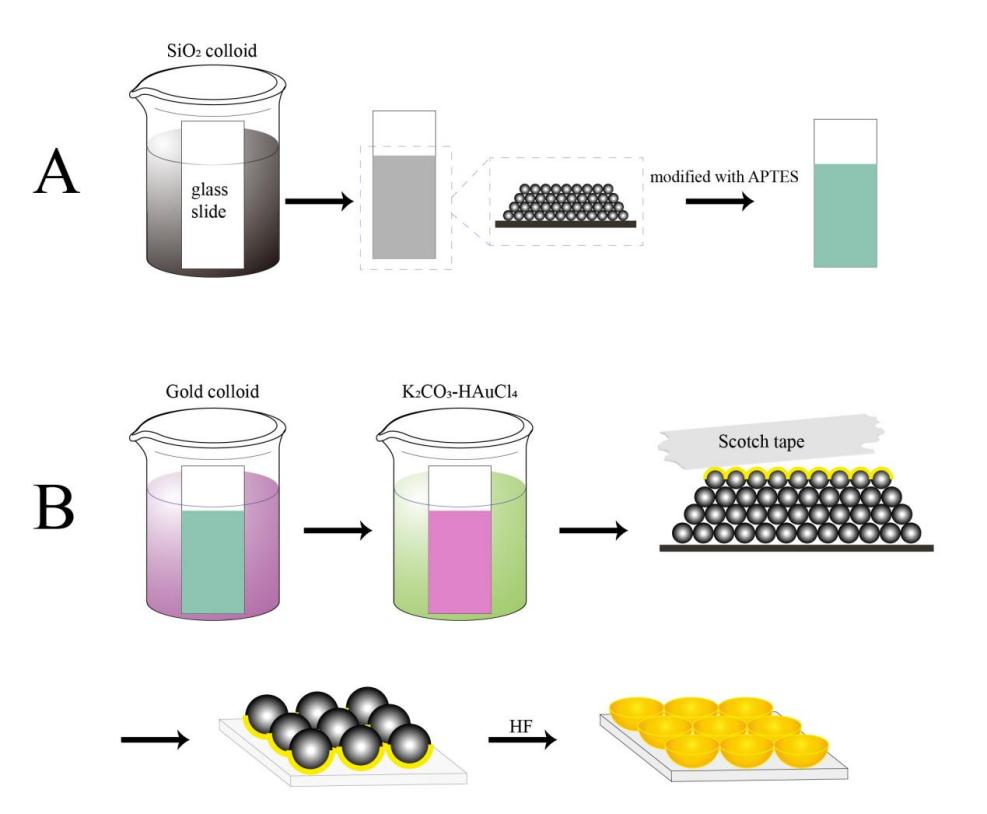


**Scheme S1.** Schematic diagram of the synthesis process of high-ordered AuNBs array. (A) The preparation of SiO_2_ colloidal crystal film and (B) the synthesis process of high-ordered AuNBs array.

To confirm the spatial electric field distribution of as-prepared AuNBs array, the finite-difference-time-domain (FDTD) simulation was performed as an effective approach under the irradiation of a beam of linearly polarized light. The complex refractive indexes of Au was adopted from the refractive index database in the simulation software package Au (Gold)-CRC. All geometric parameters were consistent with the average actual size of as-prepared samples shown in Fig. 2D. Herein, boundary conditions in Z-axis and X-, Y-axis direction were set as perfectly matched layers (PML) and periodic, respectively, in all simulations. To save computational resources while improving computational accuracy, a refined mesh grid near the structure was set as 0.48 nm in a 3D dimension 500 nm×500 nm×240 nm. The results clearly revealed that numerous “hot spots” was distributed around the edges of each pore which played a decisive role in SERS enhancement.

**
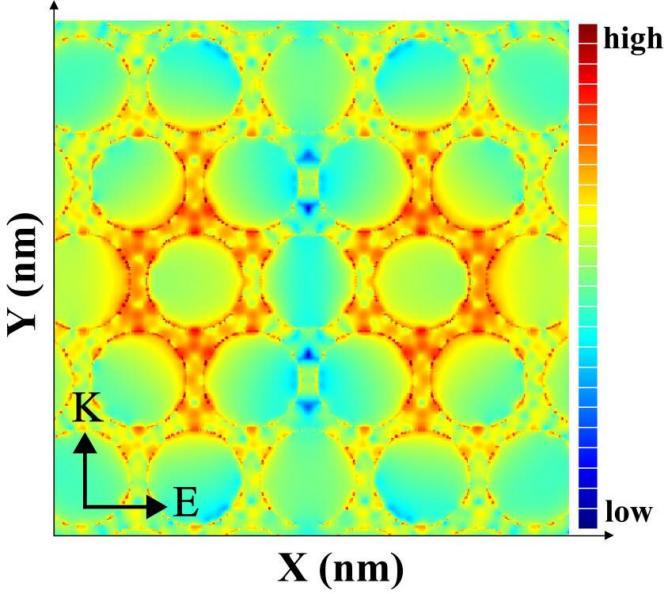
**

**Fig. S1** FDTD simulation of AuNBs array. The electric field intensity distribution images when E_z_=0 nm.

To establish the proposed pump-free and high-throughput SERS microfluidic chip, the geometry of the microfluidic device was showed in Fig. S2 followed by preparing the template corresponding to the parameter.


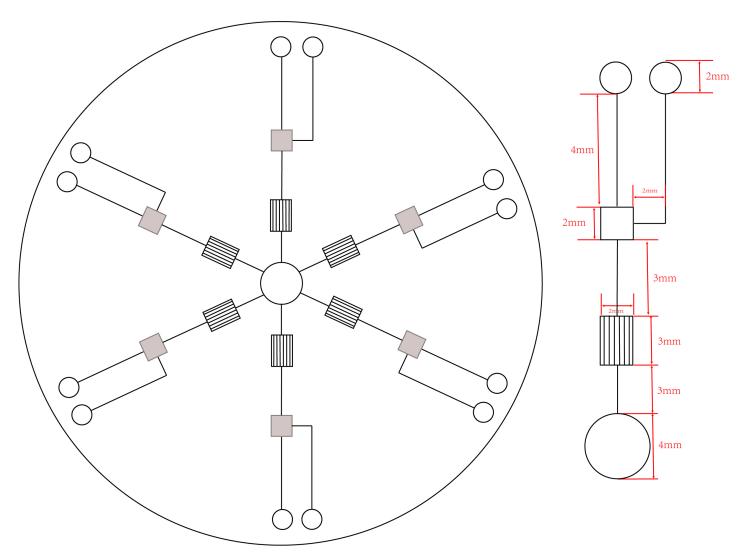


**Fig. S2** The geometry of the microfluidic device.

In order to study the hydrophilic stability, the PEG-coated SERS microfluidic chip was stored at room temperature for different days (0, 1, 3, 5 and 10 d) and then the corresponding water contact angles were measured (Fig. S2). The results clearly showed highly stable hydrophilic behaviour, as it only exhibited a slight increase in contact angle from 29° to 32° after 10 day storage.


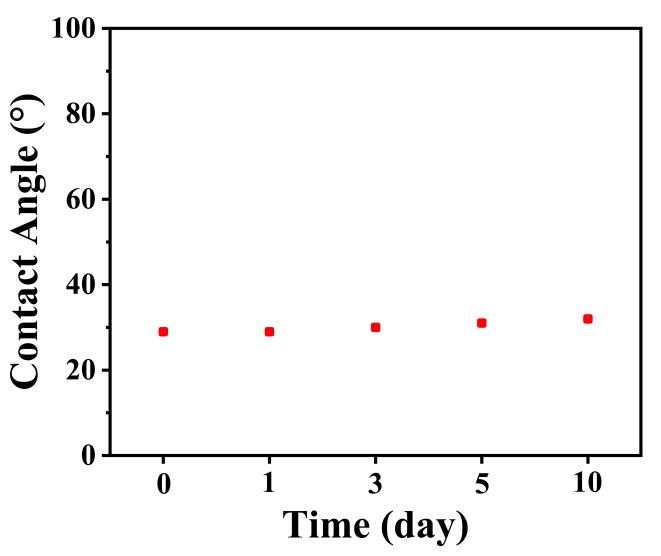


**Fig. S3** Water contact angle measurements of PEG-coated SERS microfluidic chip after storing at room temperature for different days (0, 1, 3, 5 and 10 d).

After the successful establishment of the SERS microfluidic chip, it was applied to analyze PIK3CA E542K and TP53 (Fig. S4). As shown in Fig. S4(Ⅰ), characteristic peaks at 1083 cm^-1^ and 1330 cm^-1^ could be observed when the sample contained PIK3CA E542K and TP53. Fig. S4(Ⅳ) indicated that no prominent characteristic peak could be observed when no targets existed. Fig. S4(Ⅱ) and Fig. S4(Ⅲ) demonstrated that only one characteristic peak (1080 cm^-1^ or 1330 cm^-1^) could be seen when only one target was included in the sample. Thus, the microfluidic chip could analyze PIK3CA E542K and TP53 qualitatively.


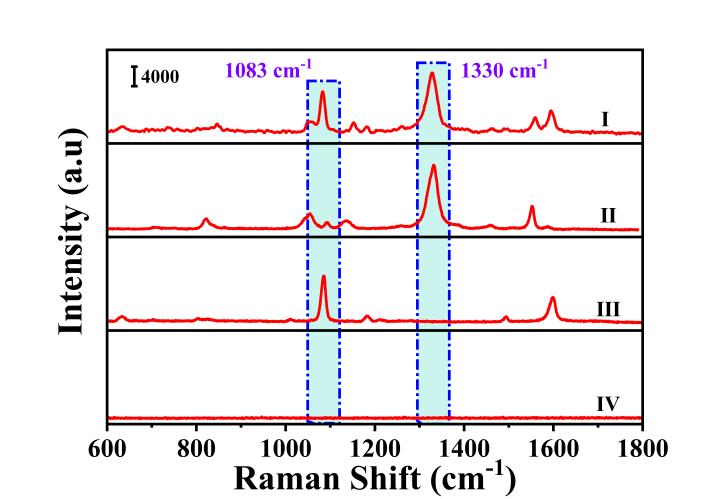


**Fig. S4** SERS spectra of three positive results and one negative result. Ⅰ: PIK3CA E542K, 100 pM; TP53, 100 pM; Ⅱ: PIK3CA E542K, 100 pM; TP53, 0 pM; Ⅲ: PIK3CA E542K, 0 pM; TP53, 100 pM; Ⅳ: PIK3CA E542K, 0 pM; TP53, 0 pM.

To verify the feasibility of the proposed SERS microfluidic chip for the quantitative determination of PIK3CA E542K and TP53, the tumor-bearing mice model was developed. The growth of subcutaneous tumors were observed and recorded using the animal living imaging system (Fig. S5). The results demonstrated that the tumors grew with time going on. Thus, the mice model was established successfully.


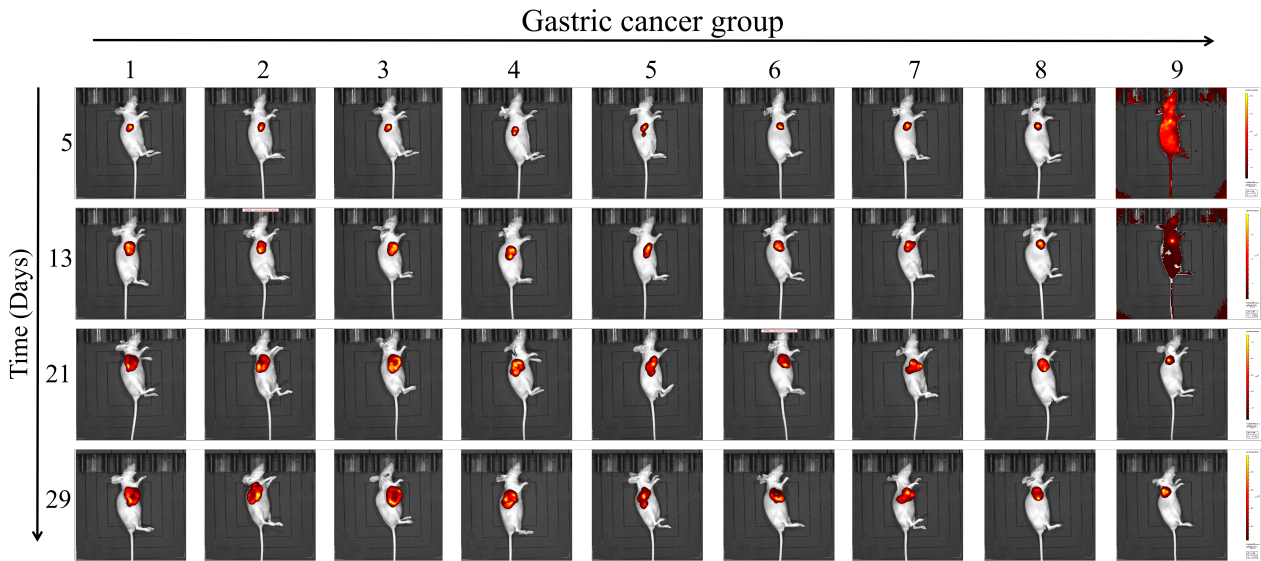


**Fig. S5** Nude mice with gastric cancer.

Under the optimized experimental conditions, quantitative analysis of PIK3CA E542K and TP53 was carried out with the concentration varying from 10 aM to 100 pM. Fig. S6A shows the SERS spectra of various concentrations of PIK3CA E542K and TP53 in PBS. The result demonstrated that the SERS intensity concomitantly enhanced with the increasing target concentrations. The data points (Fig. S6B, Fig. S6C, Fig. S6E and Fig. S6F) in the low concentration range represent a “linear” trend, and that in the high concentration range is of plateau intervals. Thus, it cannot evaluate the full-scale data in practice, leading to a narrow detection range. For this, the data set is a commonly plotted analytical response against the logarithm of concentration, which could reduce the absolute value of the data for easy calculation and not change the nature and correlation of the data. Then, a great linear relationship could be achieved between the SERS intensity at 1330 cm^-1^ and the logarithm of PIK3CA E542K concentration. The linear regression equation was y=1913.18x-1048.37, with R^2^ of 0.9839. Similarly, the regression equation for TP53 was y=1620.73x-907.23, with R^2^ of 0.9933 (Fig. S6C). Then, the limits of detection (LOD) for PIK3CA E542K and TP53 were calculated to 1.02 aM and 1.92 aM, respectively.

Subsequently, the performance of the proposed microfluidic chip in serum was also evaluated with PIK3CA E542K and TP53 spiked into mice serum to various concentrations. As shown in Fig. S6D, the SERS intensity increased with the increase in target concentrations. The linear regression equation (Fig. S6E) between SERS intensity at 1330 cm^-1^ and the logarithm of PIK3CA E542K concentrations were y=1809.22x-1116.81 (R^2^=0.9817). The linear regression equation (Fig. S6F) between SERS intensity at 1083 cm^-1^ and the logarithm of TP53 concentrations were y=1524.61x-955.53 (R^2^=0.9879). The LODs for PIK3CA E542K and TP53 were calculated to 1.26 aM and 2.04 aM, respectively. Thus, the application performance of the microfluidic chip in serum was satisfactory.


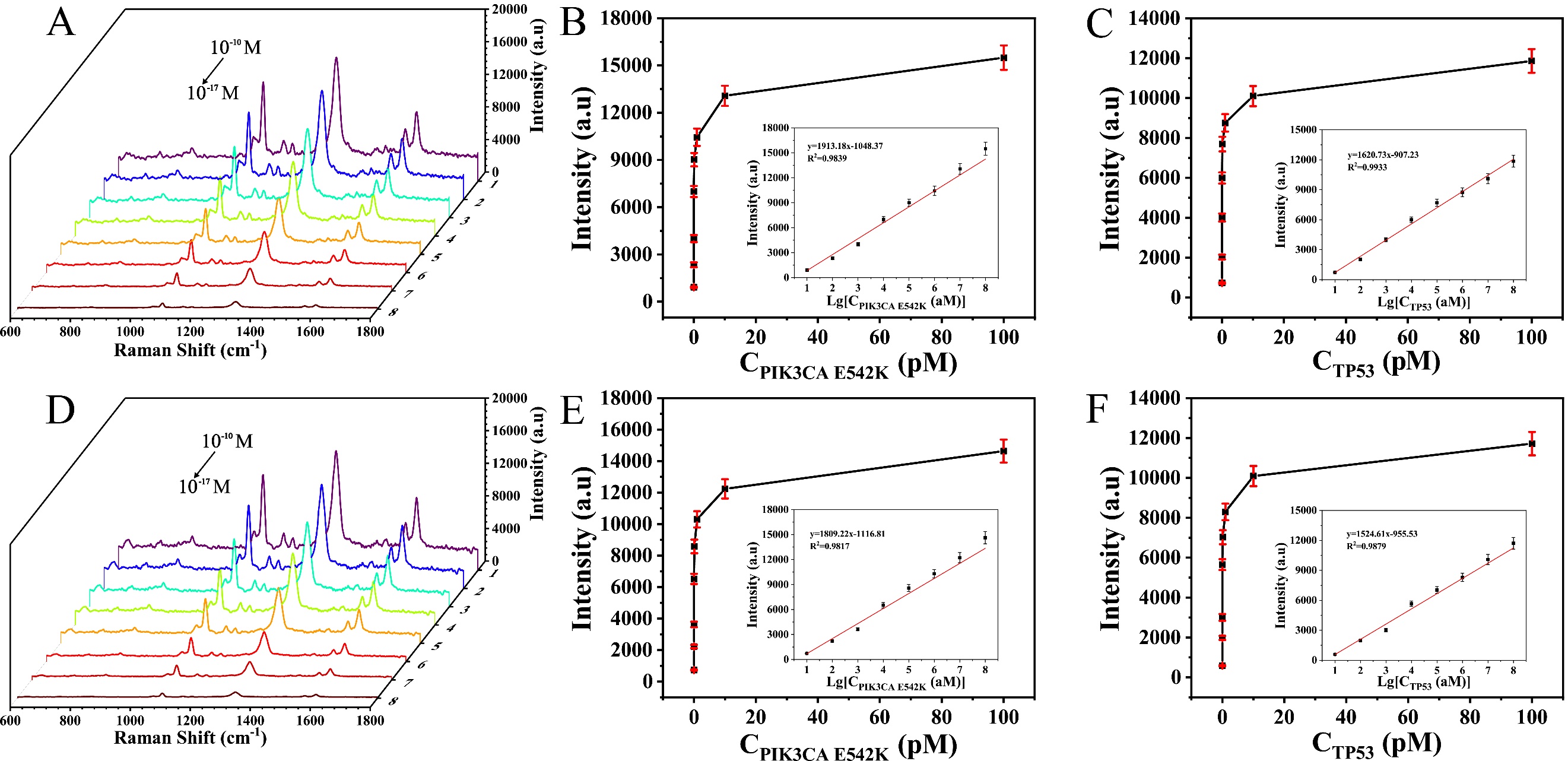


**Fig. S6** Quantification of PIK3CA E542K and TP53. SERS spectra of targets in (A) PBS and (D) mice serum with different concentrations (10 aM, 100 aM, 1 fM, 10 fM, 100 fM, 1 pM, 10 pM and 100 pM). The calibration curves of SERS intensity at 1330 cm^-1^ and 1083 cm^-1^ for the logarithm of the concentration of (B) PIK3CA E542K and (C) TP53 in PBS. The calibration curves of SERS intensity at 1330 cm^-1^ and 1083 cm^-1^ for the logarithm of the concentration of (E) PIK3CA E542K and (F) TP53 in mice serum.

Fig. S7 shows the SERS spectra of PIK3CA E542K and TP53 in tumor-bearing mice serum at different stages. The same samples were detected with qRT-PCR to verify the accuracy of SERS results. The test results of the two methods and the RSD of each group were shown in Table S3-S10. It clearly demonstrated that the proposed SERS microfluidic chip had high accuracy when used to detect real samples.


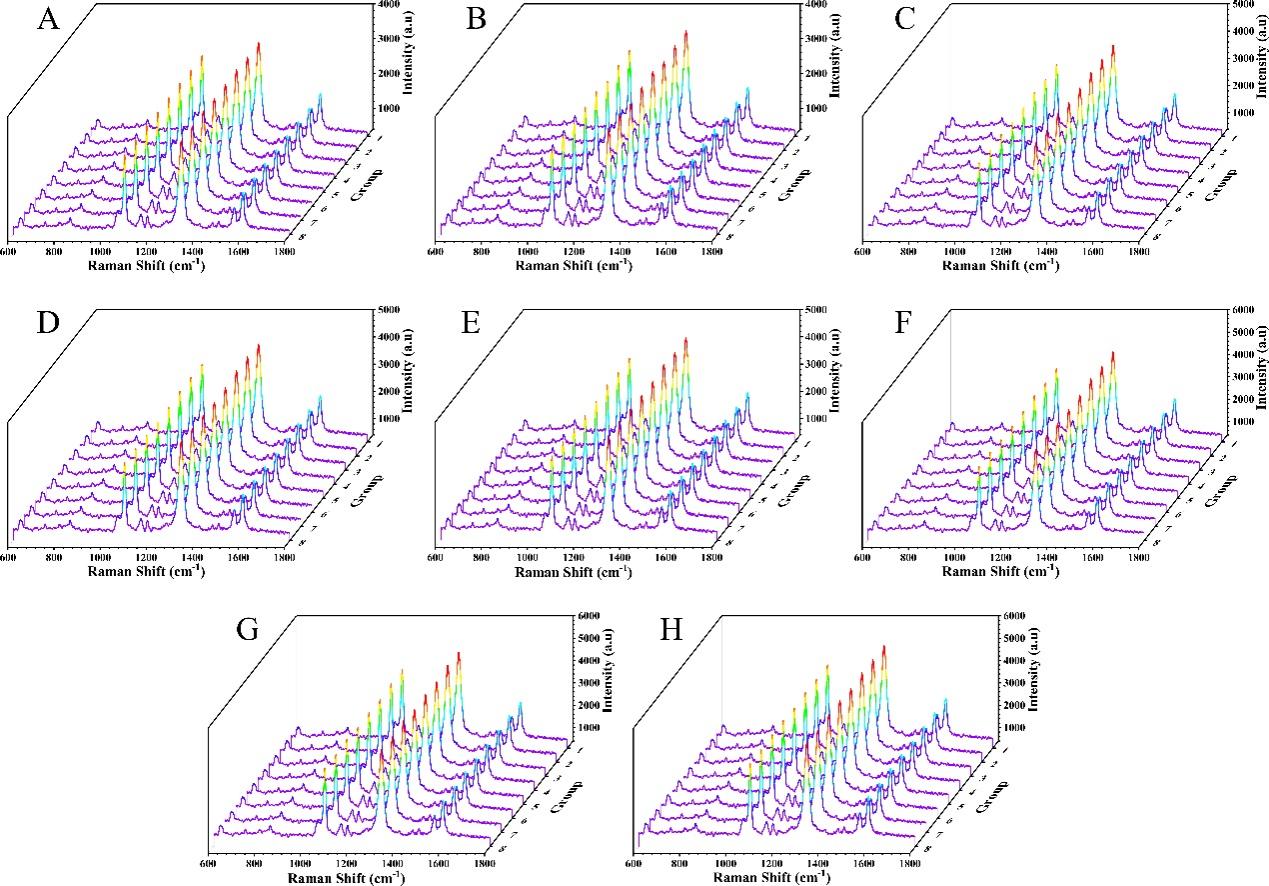


**Fig. S7** SERS spectra of PIK3CA E542K and TP53 in tumor-bearing mice serum at different stages: (A) 1 d; (B) 5 d; (C) 9 d; (D) 13 d; (E) 17 d; (F) 21 d; (G) 25 d and (H) 29 d.

**Table S1** The sequence of oligonucleotides used in the experiment

| Name | Sequence (5'-3') |
| --- | --- |
| hp1-1 | ATCCTCTCTCTAAAATCACTGAGATCCAAGTCGTAGCACCTCAGTGATTTTAG |
| hp1-2 | CATGGTGGGGGCAGCGCCTCACAACCTCATACGCCTAAGGCTGAGCGAGGTTGTGAGGCGCT |
| hp2-1 | ACTGAGGTGCTACGACTTGGATCTCAGTGATTTTAGATCCAAGTCGTAGCAC |
| hp2-2 | CACAACCTCGCTCAGCCTTAGGCGTATGAGGTTGTGAGGCGCTATACGCCTAAGGCTGAGC |
| HS-hp3-1 | HS-GTGCTACGACTTGGATCAAGCTAGACTATCCAAG |
| HS-hp3-2 | HS-GCTCAGCCTTAGGCGTATGTTCAGTACTAGCATACGCCTAA |
| HS-hp4-1 | HS-ATCCAAGTCGTAGCACCTTGGATAGTCTAGCTTG |
| HS-hp4-2 | HS-ATACGCCTAAGGCTGAGCTTAGGCGTATGCTAGTACTGAAC |
| MT1-1 | CTCAGTGATTTTAGTGAGAGGAT |
| MT1-2 | GAGGTTGTGAGGCGCTGCGCCCACCATG |
| MT3-1 | CTCTGTCATTTTAGTGAGAGGAT |
| MT3-2 | GAGGTTGAGAGGCGGTGGCCCCACCATG |
| Random | GTAGCTTATCAGACTCGACTTAGATGT |
| PIK3CA E542K | CTCAGTGATTTTAGAGAGAGGAT |
| TP53 | GAGGTTGTGAGGCGCTGCCCCCACCATG |

**Table S3** Results of SERS and qRT–PCR for real samples (1 d)

| Group | SERS (fM) | | qRT–PCR (fM) | | Relative error (%) | |
| --- | --- | --- | --- | --- | --- | --- |
|  | PIK3CA E542K | TP53 | PIK3CA E542K | TP53 | PIK3CA E542K | TP53 |
| 1 | 0.207 | 0.197 | 0.198 | 0.185 | 4.35 | 5.93 |
| 2 | 0.218 | 0.192 | 0.206 | 0.180 | 5.50 | 6.01 |
| 3 | 0.213 | 0.202 | 0.203 | 0.195 | 4.69 | 3.63 |
| 4 | 0.212 | 0.197 | 0.204 | 0.188 | 3.77 | 4.56 |
| 5 | 0.221 | 0.196 | 0.211 | 0.186 | 4.52 | 5.24 |
| 6 | 0.216 | 0.207 | 0.206 | 0.196 | 4.62 | 5.29 |
| 7 | 0.210 | 0.199 | 0.201 | 0.190 | 4.28 | 4.52 |
| 8 | 0.214 | 0.194 | 0.203 | 0.181 | 5.14 | 6.94 |

**Table S4** Results of SERS and qRT–PCR for real samples (5 d)

| Group | SERS (fM) | | qRT–PCR (fM) | | Relative error (%) | |
| --- | --- | --- | --- | --- | --- | --- |
|  | PIK3CA E542K | TP53 | PIK3CA E542K | TP53 | PIK3CA E542K | TP53 |
| 1 | 0.338 | 0.315 | 0.322 | 0.304 | 4.67 | 3.45 |
| 2 | 0.325 | 0.303 | 0.308 | 0.287 | 5.18 | 5.30 |
| 3 | 0.301 | 0.223 | 0.286 | 0.210 | 4.91 | 5.83 |
| 4 | 0.354 | 0.331 | 0.339 | 0.319 | 4.20 | 3.75 |
| 5 | 0.333 | 0.311 | 0.315 | 0.293 | 5.37 | 5.65 |
| 6 | 0.307 | 0.287 | 0.290 | 0.275 | 5.68 | 4.06 |
| 7 | 0.359 | 0.335 | 0.345 | 0.313 | 3.79 | 6.63 |
| 8 | 0.351 | 0.328 | 0.328 | 0.306 | 6.53 | 6.70 |

**Table S5** Results of SERS and qRT–PCR for real samples (9 d)

| Group | SERS (fM) | | qRT–PCR (fM) | | Relative error (%) | |
| --- | --- | --- | --- | --- | --- | --- |
|  | PIK3CA E542K | TP53 | PIK3CA E542K | TP53 | PIK3CA E542K | TP53 |
| 1 | 0.476 | 0.400 | 0.448 | 0.381 | 5.86 | 4.68 |
| 2 | 0.464 | 0.391 | 0.440 | 0.364 | 5.13 | 6.87 |
| 3 | 0.494 | 0.414 | 0.477 | 0.391 | 3.48 | 5.49 |
| 4 | 0.456 | 0.384 | 0.426 | 0.368 | 6.48 | 4.20 |
| 5 | 0.449 | 0.379 | 0.427 | 0.364 | 4.85 | 3.95 |
| 6 | 0.506 | 0.424 | 0.483 | 0.394 | 4.52 | 6.99 |
| 7 | 0.401 | 0.373 | 0.379 | 0.362 | 5.49 | 3.08 |
| 8 | 0.482 | 0.405 | 0.464 | 0.386 | 3.72 | 4.76 |

**Table S6** Results of SERS and qRT–PCR for real samples (13 d)

| Group | SERS (fM) | | qRT–PCR (fM) | | Relative error (%) | |
| --- | --- | --- | --- | --- | --- | --- |
|  | PIK3CA E542K | TP53 | PIK3CA E542K | TP53 | PIK3CA E542K | TP53 |
| 1 | 0.674 | 0.547 | 0.640 | 0.510 | 5.07 | 6.76 |
| 2 | 0.713 | 0.575 | 0.672 | 0.540 | 5.74 | 6.04 |
| 3 | 0.727 | 0.587 | 0.691 | 0.563 | 4.91 | 4.09 |
| 4 | 0.624 | 0.507 | 0.601 | 0.486 | 3.67 | 4.06 |
| 5 | 0.612 | 0.497 | 0.571 | 0.479 | 6.62 | 3.72 |
| 6 | 0.647 | 0.525 | 0.627 | 0.507 | 3.09 | 3.41 |
| 7 | 0.664 | 0.537 | 0.632 | 0.504 | 4.75 | 6.21 |
| 8 | 0.691 | 0.559 | 0.659 | 0.523 | 4.61 | 6.40 |

**Table S7** Results of SERS and qRT–PCR for real samples (17 d)

| Group | SERS (fM) | | qRT–PCR (fM) | | Relative error (%) | |
| --- | --- | --- | --- | --- | --- | --- |
|  | PIK3CA E542K | TP53 | PIK3CA E542K | TP53 | PIK3CA E542K | TP53 |
| 1 | 0.893 | 0.840 | 0.834 | 0.793 | 6.56 | 5.55 |
| 2 | 0.869 | 0.818 | 0.809 | 0.784 | 6.86 | 4.10 |
| 3 | 0.962 | 0.905 | 0.909 | 0.859 | 5.46 | 5.05 |
| 4 | 0.824 | 0.776 | 0.795 | 0.728 | 3.54 | 6.20 |
| 5 | 0.846 | 0.797 | 0.789 | 0.770 | 6.75 | 3.43 |
| 6 | 0.878 | 0.827 | 0.824 | 0.774 | 6.10 | 6.41 |
| 7 | 0.942 | 0.886 | 0.908 | 0.859 | 3.57 | 3.02 |
| 8 | 0.917 | 0.863 | 0.876 | 0.809 | 4.44 | 6.24 |

**Table S8** Results of SERS and qRT–PCR for real samples (21 d)

| Group | SERS (fM) | | qRT–PCR (fM) | | Relative error (%) | |
| --- | --- | --- | --- | --- | --- | --- |
|  | PIK3CA E542K | TP53 | PIK3CA E542K | TP53 | PIK3CA E542K | TP53 |
| 1 | 1.152 | 1.089 | 1.096 | 1.018 | 4.84 | 6.56 |
| 2 | 1.120 | 1.059 | 1.068 | 0.986 | 4.68 | 6.86 |
| 3 | 1.247 | 1.178 | 1.200 | 1.102 | 3.73 | 6.49 |
| 4 | 1.089 | 1.030 | 1.033 | 0.996 | 5.13 | 3.27 |
| 5 | 1.059 | 1.002 | 0.990 | 0.937 | 6.47 | 6.50 |
| 6 | 1.071 | 1.013 | 1.009 | 0.976 | 5.77 | 3.69 |
| 7 | 1.219 | 1.152 | 1.140 | 1.076 | 6.44 | 6.60 |
| 8 | 1.185 | 1.120 | 1.133 | 1.047 | 4.38 | 6.49 |

**Table S9** Results of SERS and qRT–PCR for real samples (25 d)

| Group | SERS (fM) | | qRT–PCR (fM) | | Relative error (%) | |
| --- | --- | --- | --- | --- | --- | --- |
|  | PIK3CA E542K | TP53 | PIK3CA E542K | TP53 | PIK3CA E542K | TP53 |
| 1 | 1.564 | 1.462 | 1.465 | 1.380 | 6.34 | 5.62 |
| 2 | 1.659 | 1.550 | 1.551 | 1.493 | 6.49 | 3.69 |
| 3 | 1.422 | 1.331 | 1.346 | 1.252 | 5.32 | 5.96 |
| 4 | 1.536 | 1.437 | 1.480 | 1.377 | 3.62 | 4.17 |
| 5 | 1.456 | 1.363 | 1.387 | 1.320 | 4.74 | 3.15 |
| 6 | 1.689 | 1.577 | 1.601 | 1.469 | 5.23 | 6.83 |
| 7 | 1.509 | 1.412 | 1.466 | 1.317 | 2.85 | 6.71 |
| 8 | 1.611 | 1.505 | 1.508 | 1.405 | 6.37 | 6.62 |

**Table S10** Results of SERS and qRT–PCR for real samples (29 d)

| Group | SERS (fM) | | qRT–PCR (fM) | | Relative error (%) | |
| --- | --- | --- | --- | --- | --- | --- |
|  | PIK3CA E542K | TP53 | PIK3CA E542K | TP53 | PIK3CA E542K | TP53 |
| 1 | 2.126 | 1.979 | 2.060 | 1.869 | 3.09 | 5.54 |
| 2 | 2.087 | 1.943 | 2.008 | 1.870 | 3.77 | 3.74 |
| 3 | 2.180 | 2.029 | 2.052 | 1.911 | 5.86 | 5.80 |
| 4 | 2.010 | 1.873 | 1.903 | 1.773 | 5.32 | 5.36 |
| 5 | 2.235 | 2.079 | 2.160 | 1.972 | 3.35 | 5.17 |
| 6 | 2.207 | 2.054 | 2.138 | 1.963 | 3.12 | 4.45 |
| 7 | 2.048 | 1.907 | 1.935 | 1.812 | 5.53 | 4.96 |
| 8 | 2.153 | 2.004 | 2.027 | 1.938 | 5.83 | 3.29 |

**Reference**

[1] Yu, J.; Lin, J.; Chen, M.; Meng, X. Y.; Qiu, L.; Wu, J.; Xi, G. C.; Wang, X. T. Amorphous Ni(OH)_2_ nanocages as efficient SERS substrates for selective recognition in mixtures. Colloids Surf. A Physicochem. Eng. Asp. 2021; 631: 127652.

[2] Liu, Q.; Yan, X.; Lai, Q.; Su, X. G. Bimetallic gold/silver nanoclusters-gold nanoparticles based fluorescent sensing platform via the inner filter effect for hyaluronidase activity detection. Sens. Actuators B Chem. 2019; 282: 45-51.

[3] Lillehoj, P. B.; Wei, F.; Ho, C. M. A self-pumping lab-on-a-chip for rapid detection of botulinum toxin. Lab Chip 2010; 10(17): 2265-2270.

[4] Xu S, Chang YY, Wu ZY, Li YR, Yuan R, Chai YQ. One DNA circle capture probe with multiple target recognition domains for simultaneous electrochemical detection of miRNA-21 and miRNA-155. Biosens. Bioelectron. 2020; 149: 111848.

[5] Ma XM, He S, Zhang HF, Li X, Xue J, Fan XL, Lu YS, Chen YT, Zhang YJ, Xu JG. Delayed full opening of bumped switchable molecular probe enables repeated generation of target analogues for mix-to-signaling determination of microRNAs. Sens. Actuators B Chem. 2021; 327: 128875.

[6] Chen CH, He RX, Zhang ZT, Chen Y. Dual-recognition-based determination of ctDNA via the clamping function of peptide nucleic acid and terminal protection of small-molecule-linked DNA. Analyst 2020; 145(23): 7603-7608.

[7] Yao YY, Zhang HD, Tian TT, Liu YX, Zhu RD, Ji J, Liu BH. Iodide-modified Ag nanoparticles coupled with DSN-Assisted cycling amplification for label-free and ultrasensitive SERS detection of MicroRNA-21. Talanta 2021; 235: 122728.

[8] Cao XW, Ge SJ, Zhou XY, Mao Y, Sun Y, Lu WB, Ran ML. A dual-signal amplification strategy based on pump-free SERS microfluidic chip for rapid and ultrasensitive detection of non-small cell lung cancer-related circulating tumor DNA in mice serum. Biosens Bioelectron. 2022; 205: 114110.
